# Metabolic versatility enables acetogens to colonize ruminants with diet-driven niche partitioning

**DOI:** 10.1093/ismejo/wraf183

**Published:** 2025-09-01

**Authors:** Qiushuang Li, Rong Wang, Xiang Zhou, Shuya Li, Shizhe Zhang, Xiumin Zhang, Wenxing Wang, Jinzhen Jiao, Peter H Janssen, Emilio M Ungerfeld, Volker Müller, Ralf Conrad, Chris Greening, Zhiliang Tan, Bo Fu, Min Wang

**Affiliations:** State Key Laboratory of Forage Breeding-by-Design and Utilization, National Engineering Laboratory for Pollution Control and Waste Utilization in Livestock and Poultry Production, Institute of Subtropical Agriculture, Chinese Academy of Sciences, Changsha, Hunan 410125, China; College of Advanced Agricultural Sciences, University of Chinese Academy of Sciences, Beijing 100049, China; State Key Laboratory of Forage Breeding-by-Design and Utilization, National Engineering Laboratory for Pollution Control and Waste Utilization in Livestock and Poultry Production, Institute of Subtropical Agriculture, Chinese Academy of Sciences, Changsha, Hunan 410125, China; State Key Laboratory of Forage Breeding-by-Design and Utilization, National Engineering Laboratory for Pollution Control and Waste Utilization in Livestock and Poultry Production, Institute of Subtropical Agriculture, Chinese Academy of Sciences, Changsha, Hunan 410125, China; College of Advanced Agricultural Sciences, University of Chinese Academy of Sciences, Beijing 100049, China; School of Environmental Science and Engineering, Wuxi University, Wuxi, Jiangsu 214105, China; State Key Laboratory of Forage Breeding-by-Design and Utilization, National Engineering Laboratory for Pollution Control and Waste Utilization in Livestock and Poultry Production, Institute of Subtropical Agriculture, Chinese Academy of Sciences, Changsha, Hunan 410125, China; College of Advanced Agricultural Sciences, University of Chinese Academy of Sciences, Beijing 100049, China; State Key Laboratory of Forage Breeding-by-Design and Utilization, National Engineering Laboratory for Pollution Control and Waste Utilization in Livestock and Poultry Production, Institute of Subtropical Agriculture, Chinese Academy of Sciences, Changsha, Hunan 410125, China; State Key Laboratory of Forage Breeding-by-Design and Utilization, National Engineering Laboratory for Pollution Control and Waste Utilization in Livestock and Poultry Production, Institute of Subtropical Agriculture, Chinese Academy of Sciences, Changsha, Hunan 410125, China; College of Advanced Agricultural Sciences, University of Chinese Academy of Sciences, Beijing 100049, China; State Key Laboratory of Forage Breeding-by-Design and Utilization, National Engineering Laboratory for Pollution Control and Waste Utilization in Livestock and Poultry Production, Institute of Subtropical Agriculture, Chinese Academy of Sciences, Changsha, Hunan 410125, China; Grasslands Research Centre, AgResearch Limited, Palmerston North 11008, New Zealand; Centro Regional de Investigación Carillanca, Instituto de Investigaciones Agropecuarias (INIA), Temuco, Vilcún 4880000, Chile; Molecular Microbiology & Bioenergetics, Institute of Molecular Biosciences, Johann Wolfgang Goethe University, Max-von-Laue Str. 9, D-60438 Frankfurt, Germany; Department of Biogeochemistry, Max Planck Institute for Terrestrial Microbiology, Karl-von-Frisch-Str. 10, D-35043 Marburg, Germany; Department of Microbiology, Biomedicine Discovery Institute, Monash University, Clayton, VIC 3800, Australia; State Key Laboratory of Forage Breeding-by-Design and Utilization, National Engineering Laboratory for Pollution Control and Waste Utilization in Livestock and Poultry Production, Institute of Subtropical Agriculture, Chinese Academy of Sciences, Changsha, Hunan 410125, China; College of Advanced Agricultural Sciences, University of Chinese Academy of Sciences, Beijing 100049, China; School of Environmental Science and Engineering, Wuxi University, Wuxi, Jiangsu 214105, China; School of Environmental and Ecology, Jiangnan University, Wuxi, Jiangsu 214105, China; State Key Laboratory of Forage Breeding-by-Design and Utilization, National Engineering Laboratory for Pollution Control and Waste Utilization in Livestock and Poultry Production, Institute of Subtropical Agriculture, Chinese Academy of Sciences, Changsha, Hunan 410125, China; College of Advanced Agricultural Sciences, University of Chinese Academy of Sciences, Beijing 100049, China

**Keywords:** ruminants, acetogen, hydrogen metabolism, comparative genomics, methane emissions

## Abstract

Enteric methane emissions are energy losses from farmed ruminants and contribute to global warming. Diverting electrons and H_2_ flow toward beneficial fermentation products would mitigate ruminal methane emissions while improving feed efficiency. Acetogens can direct H_2_ and electrons to acetate production via the Wood–Ljungdahl pathway, but methanogens have more competitive H_2_ affinities. Thus, it is unclear how acetogenesis contributes to the rumen fermentation. An analysis of 2102 globally derived rumen metagenomes from multiple ruminant species revealed that putative acetogens were phylogenetically diverse and capable of using carbohydrates or H_2_ as electron donors. The metabolic versatility of these acetogens may enable them to outcompete methanogens with lower versatility. Through animal trials, *in vitro* experiments, and DNA stable isotope probing, we verified the presence of diverse acetogens in beef cattle rumens and revealed that their niche partitioning is driven by contrasting fiber-rich and starch-rich diets. A fiber-rich diet enriched heterotrophic acetogens, which increased acetate formation while decreasing methane production. Overall, this study highlights the overlooked heterotrophy of acetogens in the rumen and their potential for mitigating enteric methane emissions.

## Introduction

Ruminant livestock harbor complex rumen microbial communities that convert low-value lignocellulosic plant material into high-value animal proteins (milk and meat) and play a crucial role in food security [1]. Rumen microbial degradation of ingested feed is a complex process performed by symbiotic microbiota, including bacteria, archaea, fungi, and protozoa, providing 70% of the ruminant’s daily energy in the form of volatile fatty acids (VFAs) [2, 3]. This incomplete microbial fermentation of feed to VFAs supports the energy requirements of the symbiotic microbiota through metabolic pathways but result in the accumulation of reduced intracellular electron carriers (like NADH and reduced ferredoxin) that must be reoxidized for fermentation to continue [4]. Molecular hydrogen (H_2_) formation is an important mechanism for electron disposal by many microbes in the rumen, and this H_2_ serves, in turn, as an energy source and electron donor for various hydrogenotrophic microorganisms. Methanogenic archaea use 70%–80% of the H_2_ for methane (CH_4_) production [5, 6], generating a greenhouse gas that contributes to global warming and resulting in energy loss of the feed ingested [7, 8]. Redirecting electrons and H_2_ flow away from methanogenesis to alternative sinks, such as acetogenesis, nitrate reduction, and fumarate reduction, offers the potential to simultaneously mitigate CH_4_ emissions while improving animal energy efficiency [9, 10].

Acetogens are a specialized group of anaerobic bacteria that can use the reductive Wood–Ljungdahl (WL) pathway to reduce CO_2_ as a carbon source for cell biosynthesis, and as an electron sink when using H_2_ and other compounds as energy sources [11, 12]. During reductive acetogenesis, two molecules of CO_2_ are reduced to methyl and carbonyl groups, which are then combined to form one molecule of acetyl-CoA; acetate synthesis is catalyzed by acetyl-CoA synthase and requires the incorporation of eight reducing equivalents (eight electrons) [13]. In comparison, the same number of reducing equivalents is used by methanogens to reduce one mol CO_2_ to one mol CH_4_. Therefore, enhanced acetogen activity would benefit the ruminant host by resulting in the production of absorbable acetate rather than net energy loss as CH_4_. However, the threshold of dissolved H_2_ concentration for reductive acetogenesis is much higher than that for methanogenesis, causing reductive acetogenesis to be thermodynamically outcompeted by methanogenesis due to a lower affinity for H_2_ [5, 6]. Acetogens exhibit remarkable metabolic flexibility: as well as growing autotrophically (using H₂/CO₂), they can grow heterotrophically (fermenting diverse substrates like sugars and organic acids), or mixotrophically by combining these strategies [11, 14], which enables electron disposal via acetate synthesis during organic substrate metabolism, potentially circumventing direct competition with methanogens for H₂. How these metabolic strategies sustain their survival and coexistence with methanogens in this H₂-limited niche remains unclear.

**Figure 1:**
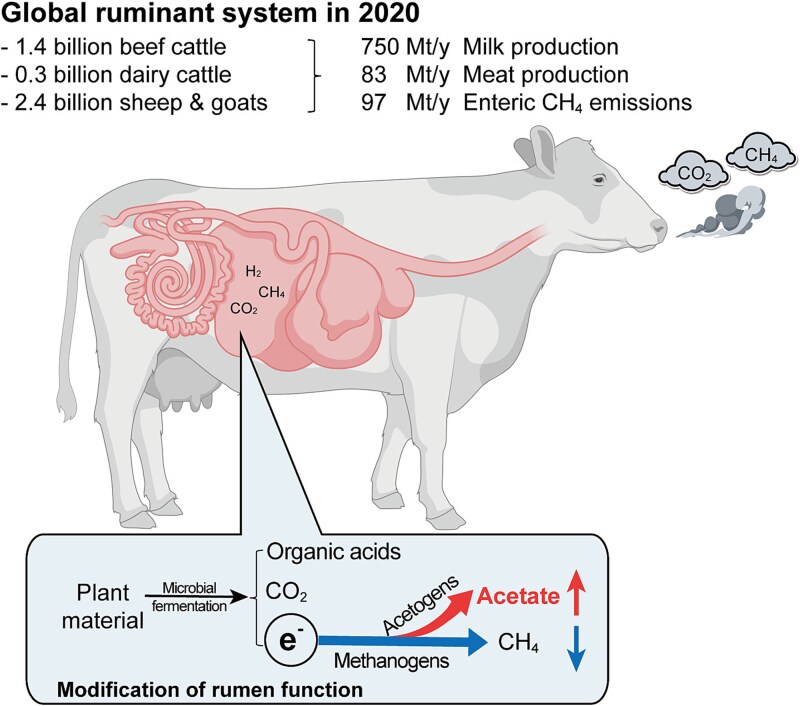
Ruminal methane (CH_4_) formation and redirection of electrons away from methanogenesis for reductive acetogenesis in ruminant livestock. The data presented in this figure were derived from Food and Agriculture Organization of the United Nations (https://www.fao.org/faostat/en/#data/QCL). The cattle model was made using BioRender.

Here, we addressed whether acetogens are present, active, and adapted to the rumen microecosystem. Previous research has provided metagenomic evidence that acetogens inhabit the rumen and use diverse mechanisms to harvest energy, with their abundance and activity elevated both in ruminants selectively bred for low CH_4_ emissions and in animals exposed to methanogenesis inhibitors [12, 15, 16]. Still, a holistic understanding of their biodiversity, metabolic features, and ecological roles is lacking. To address this knowledge gap, we combined metagenomic approaches, stable carbon isotope fractionation, *in vivo* ruminant and *in vitro* rumen fermentation trials, thermodynamic modeling, and stable isotope probing (SIP). This revealed that ruminant acetogens are abundant, active, phylogenetically, and metabolically diverse members of rumen microbiomes. Moreover, we provide direct SIP-based evidence that they can mediate both hydrogenotrophic and heterotrophic acetogenesis, with the latter enriched in Xiangxi beef cattle fed with a fiber-rich diet versus a starch-rich diet. Enrichment of acetogens results in increased acetate formation concomitant with decreased methanogenesis. The projected increase of acetate produced via reductive acetogenesis in the rumen microecosystem provides solutions to mitigate CH_4_ emissions while enhancing the sustainability of the ruminant system for food production (Fig. 1).

## Materials and methods

### Metagenomic datasets for the ruminant digestive tract

**Figure 2:**
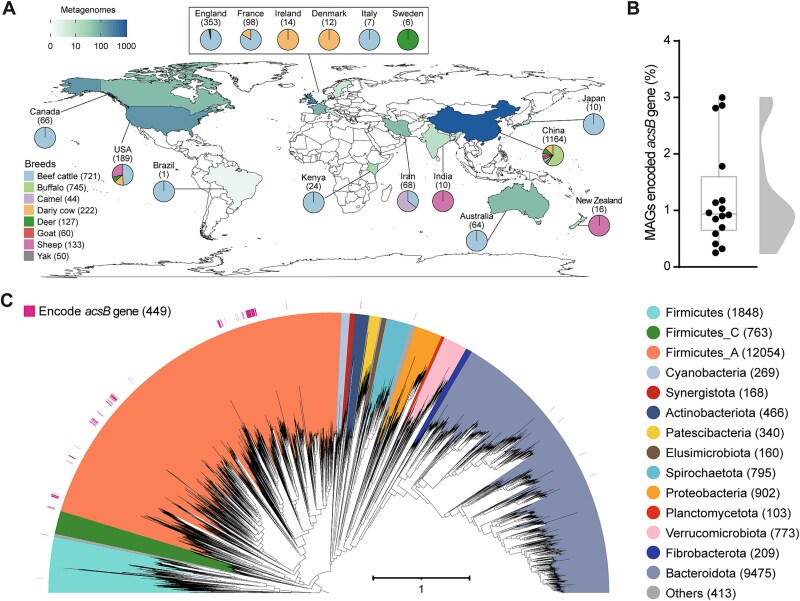
Origins of ruminant metagenomic samples and strain-level classifications. (A) Geographic distribution of ruminant metagenomic samples collected; pie plots show the proportion of metagenomic samples obtained from each ruminant breed in each country; numbers in brackets represent the number of metagenomic samples obtained from each country or ruminant breed. Detailed information on all samples is provided in the Supplementary File S1. (B) Percentages of MAGs containing *acsB* genes in individual studies. (C) A maximum-likelihood phylogenetic tree of 28 738 bacterial MAGs compiled for this study. Clades are colored by the GTDB phylum annotation, with outer circles depicting genomes encoding the *acsB* gene, a key enzyme in the Wood-Ljungdahl pathway. The tree was produced from concatenated protein sequences using IQ-TREE and subsequently drawn using iTOL. The scale bar indicates the average number of substitutions per site.

Publicly available metagenomes were compiled for 2102 ruminant digestive tract samples collected from seven ruminant species (including beef cattle, buffalo, camel, dairy cow, deer, goat, sheep, and yak) in 20 studies across 16 countries around the world (Fig. 2A, Supplementary File S1, and references therein). Of these, 1944 metagenomic data sets, which have published metagenome-assembled genomes (MAGs), were obtained from previous publications (details in Supplementary File S1). A further 158 metagenomes without binning were downloaded from the Sequence Read Archive (SRA) at the National Center for Biotechnology Information (NCBI) or European Nucleotide Archive (ENA), and then the microbial genomes were constructed following our workflow (Supplementary Fig. S1). Only metagenomes accessible before 31 September 2023 were considered.

### Metagenome assembly and binning

Metagenomic paired-end raw reads were quality-controlled by trimming primers and adapters and filtering out artifacts and low-quality reads using the Read_QC module within the metaWRAP pipeline (v1.3.2; --skip-bmtagger) [17], and host reads were removed according to different sample sources by bowtie2 (v2.3.5.1) [18]. For each study, clean reads were individually assembled using MEGAHIT (v1.2.9; --min-contig-len 1000) [19], and then contigs were binned using the binning module (--metabat2 --metabat1 --maxbin2 --concoct) and consolidated into a final bin set using DAS Tool (v1.1.6) [20]. All produced bins in a study were aggregated and dereplicated to a non-redundant set of strain-level MAGs using dRep [21] (v3.3.0; -comp 50 -con 10) at 99% average nucleotide identities (ANI). Completeness and contamination of MAGs were evaluated using CheckM (v1.2.0) [22], with genomes meeting completeness ≥50% and contamination <10% classified as medium-quality, whereas those with completeness >90% and contamination <5% were designated as high-quality.

### Taxonomic assignment, functional annotations, and phylogenetic analysis

The taxonomic classification of each MAG was assigned based on the Genome Taxonomy Database via the classification workflow of GTDB-TK (v2.1.1, R07-RS207) [23]. Carbohydrate-active enzymes (CAZymes) [24] of each MAG were annotated using HMMER [25] and DIAMOND [26] to obtain CAZyme annotations through three approaches integrated in dbCAN3 [27]. A query was acceptable only if all three approaches (DIAMOND, dbCAN_sub, and dbCAN) reported the same match. KO functional orthologs (KOs) were annotated using METABOLIC (v4.0) [28]. Hydrogenases mediating H_2_ production and consumption, namely [NiFe]-, [FeFe]-, and [Fe] hydrogenases, were identified with the HydDB database [29] using DIAMOND [26] with an e-value threshold of 1e-50, one maximum target sequence per query, and subsequent filtered (length of amino acid >40 residues, sequence identity >60%). The gene abundance in the community was estimated using GCompip [30]. Detailed parameters are provided in the Supplementary Materials and Methods.

The maximum-likelihood phylogenetic trees of 28 738 bacterial genomes and 75 genomes of putative acetogens were constructed based on multiple sequence alignments produced by GTDB-TK using IQ-TREE [31] (v2.2.2.7; −m MFP -nt 200 -bb 1000 -redo -mredo). The genome-guided criteria for identifying putative acetogenic bacteria according to a published study are as follows [32]: they must possess the key genes encoding enzymes in the WL pathway, including *acsA* and *acsB* for carbon-monoxide dehydrogenase/acetyl-CoA synthase (CODH/ACS), *fhs* for formate-tetrahydrofolate ligase (FTHFS), *fchA* for methenyltetrahydrofolate cyclohydrolase/methylenetetrahydrofolate dehydrogenase complex (MC/MD), *metF* for methylenetetrahydrofolate reductase (MR), and *acsE* for methyltransferase (MT). The presence of formate dehydrogenase (FDH) was not obligatory for the classification, because some acetogens lack *fdh* genes [32, 33]. Each phylogenetic tree of enzymes involved in the WL pathway was built as follows (the FolD was not analyzed due to the inadequate number of sequences). Firstly, amino acid sequences were retrieved from genomes of putative acetogen and then aligned using MAFFT [34] (v7.520). Secondly, alignments were further trimmed using TrimAl [35] (v1.4.rev22; −gappyout -htmlout). Then, maximum-likelihood trees were constructed using IQ-TREE [31] (−m MFP -nt 200 -bb 1000 -redo -mredo). Finally, all the produced trees were visualized and beautified in the Interactive Tree of Life (iTOL; v6) [36]. Detailed information on genomic context, conserved motifs, and protein structure identification is provided in the Supplementary Materials and Methods.

### Animal experimental design, rumen sample collection, and measurement

All animals involved in the experiment were cared for following the Animal Care and Use Guideline of the Animal Care Committee, Institute of Subtropical Agriculture, the Chinese Academy of Sciences, Changsha, China, with all animal experimental procedures approved by the Committee (approval number: ISA-W-201901).

A total of 20 local breed Xiangxi beef cattle (initial body weight 135 ± 10.7 kg) were randomly divided into two dietary treatments that lasted for 300 d. The high-fiber diet was formulated to have a 30% concentrate on a dry matter (DM) basis, whereas the high-starch diet was formulated to have a 90% concentrate (DM basis; Supplementary Table S1), of which starch was mainly from barely meal. All the cattle were fed twice daily at 7 a.m. and 5 p.m. and had free access to drinking water.

Rumen samples were collected at 0, 2.5, and 6 h after the morning feeding on two consecutive days at the end of the experiment. About 500 ml of rumen content was collected by a stainless-steel stomach tube with a rumen vacuum sampler, and the first 150 ml of rumen contents were discarded to avoid saliva contamination [37]. About 20 ml of sampled rumen contents was used for immediately measuring pH with a portable pH meter (Starter 300; Ohaus Instruments Co. Ltd., Shanghai, China). Three 50-ml subsamples were immediately frozen in liquid N_2_ and stored at −80°C for microbial DNA extraction. Three 5-ml subsamples of rumen contents were collected and centrifuged at 12000 × *g* for 10 min at 4°C. A 1.5 ml aliquot of supernatant was acidified with 0.15 ml of metaphosphoric acid (25%, w/v) and stored at −20°C for subsequent measurement of fermentation products. Individual volatile fatty acids (VFA) concentrations were analyzed by gas chromatography (Agilent 7890A, Agilent Inc., Palo Alto, CA) as previously described [38]. The detailed procedures of ^13^C ratio measurement in individual VFAs, microbial DNA extraction, metagenome sequencing, and bioinformatic analysis are provided in the Supplementary Materials and Methods.

### Measurement of microbiome activity through *in vitro* ruminal fermentation

An *in vitro* experiment was conducted to compare the fermentative activities, including gas and VFA production, of rumen microbiomes derived from fiber-rich and starch-rich cattle (n = 6), by individually incubating starchy, fibrous, or rice straw substrates, following a previously established procedure [39]. Briefly, rumen contents were collected before the morning feeding using a stainless-steel stomach tube, filtered through five layers of cheesecloth, and then mixed with pre-warmed McDougall’s buffer (volume ratio of 1 to 4) to prepare the buffered rumen fluid [38]. Buffered rumen fluid (60 ml) was delivered into 150-ml bottles containing 1 g substrate and sealed. All these procedures were performed under a stream of CO_2_. *In vitro* batch cultures were incubated at 39.5°C for 48 h. The pressure inside each bottle was measured and recorded every 1 min. When the pressure inside any bottle exceeded 10 kPa, the three-way solenoid valve on that bottle opened to release the excess gas, and CH_4_ and H_2_ concentrations were determined through gas chromatography (Agilent 7890A, Agilent Inc., Palo Alto, CA) [37]. Methane production was then calculated by using the equation as previously described [40]. Samples from the liquid phase were collected from each bottle after finishing the incubations, snap frozen in liquid N_2,_ and stored at −80°C for further analysis of fermentation end products. Solid residues were filtered into pre-weighed Gooch filter crucibles and dried at 105°C for 24 h, and weighed to determine the degradation of incubated substrates.

### DNA-stable isotope probing experiments and microbial DNA extraction

The experiment setup consisted of 120 ml serum bottles containing 8 ml of mixed rumen liquid obtained from the Xiangxi beef cattle experiment described above and 42 ml of basic salt medium (initial pH of 7.0), then the bottles were closed with black rubber stoppers [41]. The headspace in the bottle was amended with ^13^CO_2_-H_2_ or ^13^CO_2_-N_2_ (80/20, v/v) with the headspace pressures of 0.15 MPa, with each treatment including six biological replicates. Sodium 2-bromoethanesulfonate (BES) was supplemented at 10 mM to inhibit methanogenesis, followed by bottle incubation at 37°C, with all experiments conducted in triplicate technical replicates. Headspace gas samples were taken from the headspace during the incubation at 3-day intervals to measure the concentrations of H_2_ and CO_2,_ and 2 ml of liquid samples were collected for analysis of VFAs concentration. The bottles were refilled with the same gas mixture to 0.15 MPa when the headspace pressure decreased below 0.11 MPa at day 15. The incubation was performed for 28 days, and then the samples were collected and stored frozen at −20°C for later analyses.

Microbial DNA was extracted following the protocol outlined by a previously published method based on repeated sand beating plus column methodology [42, 43], and the extracted DNA was subsequently purified using phenol/chloroform/isopentyl alcohol (25:24:1 vol/vol/vol, Solarbio Co., Shanghai, China). The integrity of the extracted DNA was evaluated through electrophoresis on 0.8% agarose gels, and the DNA concentration and quality were determined using an ND-2000 spectrophotometer (NanoDrop Technologies, Wilmington, DE). All DNA samples were stored at −80°C until further analyses. Gradient fractionation was performed according to a previously published method [44]. DNA (2 mg) was combined with CsCl (1.72 g/ml) and gradient buffer (100 mM Tris–HCl pH 8.0, 100 mM KCl, 1 mM EDTA) in an ultracentrifugation tube (Quick-Seal Centrifuge Tubes 13 × 51 mm, 5.1 ml, Beckman Coulter, Pasadena, California) and ultra-centrifuged at 2580000 × *g* (Optima XPN-100 Ultracentrifuge, Beckman Coulter, USA) under vacuum at 20°C for 44 h. Gradient fractionation resulted in 15 DNA fractions of ~200 μl each, whose density was measured with a refractometer (AR200, Reichert Technologies, New York, USA). DNA was precipitated from the CsCl with polyethylene glycol solution (30% PEG6000, 1.6 M NaCl), washed with 70% ethanol, and eluted in 30 μl DES solution. The integrity of the extracted DNA was evaluated through electrophoresis on 0.8% agarose gels, and DNA concentrations and qualities were determined by a spectrophotometer (NanoDrop 2000, Thermo Fisher Scientific, MA, USA). All DNA samples were stored at −80°C until further analyses. The unlabeled substrate incubations were used as controls to determine the expected position of labeled DNA in the CsCl density gradients. All samples were sequenced on the HiSeq X System (Illumina, San Diego, CA, USA) with pair-end 150 bp reads. The bioinformatics analysis processes followed the pipeline described previously.

### Statistical analyses

Generalized linear models were used to analyze metabolite concentrations and production using the SPSS 21.0 software (SPSS Inc., Chicago, IL, USA). For models incorporating sampling time, a linear mixed model was applied with treatment, sampling time, and their interaction as fixed effects, and the animal as a random factor. The Wilcoxon rank-sum test in the JMP Pro software (JMP Pro version 13.2.1, SAS Institute Inc., SAS Institute, Cary, NC, USA) was employed to analyze the relative abundance of the MAGs. All *P* values were adjusted for False Discovery Rate (FDR) using the Benjamini-Hochberg method, considering *P* < .05 as statistically significant.

## Results and discussion

### Acetogens are widely distributed in rumen microbiomes

We collected 2102 publicly available metagenomes from eight ruminant species, spanning 20 studies from 16 countries (Fig. 2A and Supplementary File S1), and generated metagenome-assembled genomes (MAGs) from these to explore the prevalence of reductive acetogens. The *acsB* gene encodes acetyl-CoA synthase, an enzyme exclusive to the WL pathway, which is conserved among all known isolated acetogens, and has been widely recognized as a marker for acetogens in metagenomic studies [12, 45, 46]. We therefore used the *acsB* gene as a genetic marker of acetogens that use the WL pathway. MAGs harboring *acsB* genes were detected in almost all metagenomes (16 of 20 studies) and constituted a median of 0.9% (ranging from 0.3% to 3.0%) among all the MAGs in the 16 studies of metagenomes that contained *acsB* genes (Fig. 2B and Supplementary File S1). Meanwhile, MAGs harboring *acsB* genes were also widely distributed in the gastrointestinal tracts of the animal hosts the MAGs originated from, which were beef and dairy cattle, and six other ruminant species, with an average proportion by ruminant species of 1.4% (ranging from 0.7% to 2.9%; Supplementary Table S2). This consistent presence further confirms that acetogens are persistent ruminal community members, with prevalence ranges comparable to methanogens.

We integrated the MAGs from all the studies and dereplicated them with a 99% average nucleotide identity cutoff to avoid analyzing duplicate genomes from this point onward. A total of 29 247 non-redundant genomes (28 738 bacteria and 509 archaea) were retained, including 10 345 high-quality genomes and 18 902 medium-quality genomes clustered at the strain level [47] (Fig. 2C and Supplementary File S2), which constitutes a global ruminant microbial genome dataset. The 29 247 strain clusters in our integrated database belonged to 35 known phyla (31 bacterial and 4 archaeal; Fig. 1B and Supplementary File S2) based on the Genome Taxonomy Database (GTDB; version R07-RS207) [23]. Bacteria from the *Firmicutes*_A (*n* = 12 054), *Bacteroidota* (*n* = 9475), and *Firmicutes* (*n* = 1848) made up most of our database (81.5%). The two major archaeal phyla were *Methanobacteriota* (*n* = 315) and *Thermoplasmatota* (*n* = 119, Supplementary File S2). This global ruminant microbial dataset has a similar structure to other published ruminant microbial genome datasets [48–50]. Among these curated bacterial genomes, 449 (1.6%) MAGs, including 153 high-quality and 296 medium-quality MAGs (Supplementary File S3), harbored *acsB* genes, encoding the α subunit of CODH/ACS synthase [51]. These *acsB*-containing MAGs originated from 11 bacterial orders within four phyla, most prominently represented by the orders *Oscillospirales* and *Lachnospirales* within *Firmicutes*_A (Fig. 2C and Supplementary File S3). This suggests that bacterial genomes harboring reductive acetogenesis are widely distributed and span diverse orders in ruminant microbiomes.

### Acetogens are phylogenetically diverse with the highly conserved function of reductive acetogenesis

**Figure 3:**
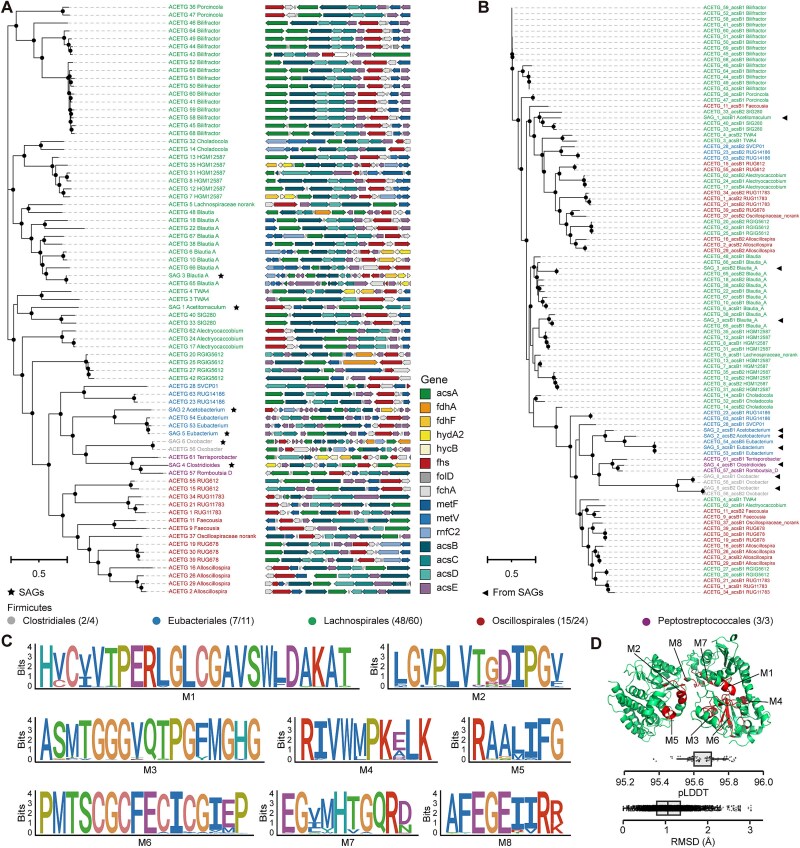
Genetic analysis of genomes derived from putative acetogens and the AcsB protein sequences. (A) Phylogenetic analysis of the genomes obtained from this study and six single amplified genomes (SAGs) according to previous research [32]. Genomes are colored based on their phylogenetic affiliation at the order level, and the genomic context of 15 genes involved in the Wood–Ljungdahl pathway is shown beside the corresponding genome. (B) Phylogenetic analysis of the AcsB sequences. The sequences were obtained from 75 genomes. (C) Eight amino acid motifs of AcsB by Multiple Em for Motif Elicitation (MEME) with default parameters. The size of the graphic character corresponding to each residue is directly proportional to its frequency at that location. (D) Tertiary structures representing the catalytic subunit of CODH/ACS encoded by the *acsB* gene from ACETG_43 MAG (*Bilifractor* sp.), which was modeled using AlphaFold2 in ColabFold and subsequently visualized with Pymol. The predicted local distance difference test value (pLDDT) of each structure and the RMSD for each pair of structures are presented below. Bootstrap values of >80% are indicated as black circles at the nodes, and the scale bar indicates the average number of substitutions per site. M1 to M8: motif1 to motif8.

A total of 69 MAGs of putative acetogens were further selected based on strict criteria involving the presence of six genes encoding key enzymes of the WL pathway (Supplementary Figs. S1-S3 and File S4) [32]. To ensure a comprehensive analysis, we integrated six single-amplified genomes of ruminal acetogens (SAGs; Supplementary Table S3) according to previous research derived from pure culture as gold-standard references [32], and combined them with existing genomic resources to establish a curated database comprising 75 genomes for further in-depth analyses. Phylogenetic profiling revealed that these putative acetogens belong to 26 genera, with the dominant orders being *Lachnospirales* (n = 48; mainly *Bilifractor*, previously known as *Eubacterium*) and *Oscillospirales* (n = 15; mainly the probable genus RUG11783) of the phylum *Firmicutes_*A (Fig. 3A). Previous studies have also shown that ruminal acetogens are primarily affiliated with *Lachnospiraceae* in the phylum *Firmicutes* [52, 53]. Numerous MAGs belonged to uncultivated genera, such as RGIG5612 and HGM12587, representing potentially novel acetogens. Further genome context analysis showed that the organization of genes encoding key enzymes, such as CODH/ACS and formate-tetrahydrofolate ligase (FTHFS) of the WL pathway, was similar across genera, with genes encoding the same complex generally clustered within individual genomes (Fig. 3A), in line with previous reports [54, 55]. The *fdhA*/*fdhF* genes encoding the dispensable formate dehydrogenase were co-localized with the cluster of the WL pathway. Some genomes contained *hycB*, as well as *fdhF* and *hydA2* (Fig. 3A). This triad appears to constitute an H_2_-dependent CO₂ reductase, which appears to often be dispensable for acetogens in the formate-rich and low-H_2_ partial pressure environments of the rumen. Overall, the putative acetogens are phylogenetically diverse with distinct clusters of key genes encoding enzymes involved in the WL pathway across genera in ruminant microbiomes.

The CODH/ACS protein, a key functional marker of acetogens, was further studied through analysis of 102 *acsB* sequences retrieved from the MAGs of putative acetogens (Fig. 3B). Most sequences (n = 60) were classified within *Lachnospirales*, primarily the genus *Bilifractor*. Phylogenetic analysis of the inferred translations revealed that the AcsB sequences from the same orders were clustered into different clades (Fig. 3B). All the AcsB sequences were subjected to multiple sequence alignments and at least eight conserved motifs were found (Fig. 3C). The predicted tertiary structures of the catalytic subunit of the CODH/ACS protein encoded by the *acsB* gene had pairwise local distance distribution test (pLDDT) values greater than 95% (mean = 95.7%, standard deviation [SD] = 0.10; Fig. 3D). Further comprehensive two-by-two structural alignment of the proteins encoded by the *acsB* gene yielded Root-Mean-Square Deviation (RMSD) values around 1 Å (mean = 1.09 Å, SD = 0.449; Fig. 3D), indicating strong conservation of 3D structure. We then analyzed the additional 13 genes encoding key enzymes of the WL pathway, including other subunits of the CODH/ACS synthase (*acsA*, *acsC*, and *acsD*), formate dehydrogenase (*fdhA*, *fdhF*, *hydA2*, and *hycB*), FTHFS (*fhs*), methenyltetrahydrofolate cyclohydrolase (*fchA*), methylenetetrahydrofolate reductase (*metF*, *metV*, *rnfC2*), and methyltransferase (*acsE*). Their phylogenies showed a similar pattern to *acsB*, with highly conserved motifs and primary structures across these MAGs (Supplementary Figs. S4-S16, Files S5, and S6). Altogether, these genomes showed remarkable conservation and clustering of each gene within the WL pathway, indicating a highly conserved function of reductive acetogenesis among diverse acetogen taxa.

### Acetogens harbor the flexibility to incorporate electrons from oxidizing carbohydrates and molecular hydrogen

**Figure 4:**
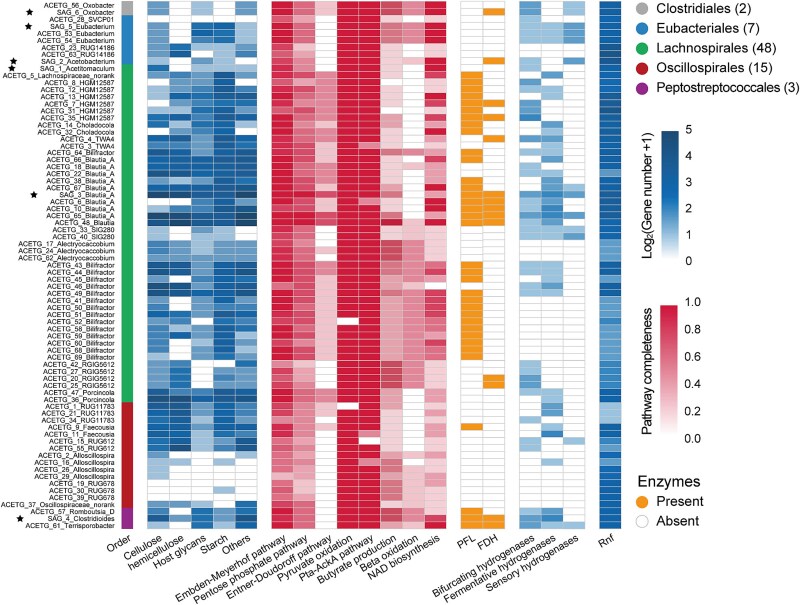
Identification of diverse metabolic features in the genomes of putative acetogen. The heatmaps indicate the number of genes encoding carbohydrate-degrading enzymes; the completeness of metabolic pathways involved in fermentative glucose degradation; the presence of genes encoding FDH and PFL; the number of genes encoding hydrogenases; and the number of genes encoding Rnf/Ech complexes. Analysis was performed based on 75 genomes, comprising 69 MAGs from this study and six previously published SAGs (indicated with stars). PFL, pyruvate formate-lyase; FDH, formate dehydrogenase.

The genes involved in the carbon, hydrogen, and energy metabolism of the putative acetogens were further analyzed to understand their potential function in the rumen. Among the 75 genomes we had selected for further analysis, 72 harbored genes encoding carbohydrate-degrading enzymes for cellulose (GH3 and GH94), hemicellulose (GH2 and GH43), host glycans (GH18 and GH36), and starch (GH13 and GH77; Fig. 4 and Supplementary File S7). Although acetogens are generally thought to use the simpler biodegradation products of most natural polymers, such as cellulose and lignin [11, 56–59], these results suggest that the acetogens themselves have the ability to degrade complex polysaccharides in ruminants, and efforts to cultivate these novel acetogens would help confirm this ability.

Further analysis of polysaccharide-degrading pathways revealed that all the 75 genomes harbored the genes encoding the Embden-Meyerhof pathway (glycolysis) to oxidize glucose for pyruvate production. The detection of several genes encoding pyruvate dehydrogenase and pyruvate-ferredoxin oxidoreductase across 73 of the 75 genomes suggest they can use pyruvate to generate acetyl-CoA and reduced ferredoxin [60, 61]. Genes encoding pyruvate formate-lyase (PFL) were detected in 35 genomes, suggesting their ability to cleave pyruvate into formate and acetyl-CoA [62]. Additionally, 74 of the 75 genomes encoded phosphotransacetylase and acetate kinase genes to obtain ATP by converting acetyl-CoA into acetate (Fig. 4, Supplementary File S7, and Fig. S17). Furthermore, all the genomes also contained genes involved in the pentose phosphate pathway to produce glyceraldehyde 3-phosphate and pyruvate from glucose 6-phosphate. Among these, 44 genomes belonging to *Lachnospirales*, *Oscillospirales*, and *Clostridiales* also harbored genes coding for the Entner-Doudoroff pathway, which rapidly converts glucose 6-phosphate into glyceraldehyde 3-phosphate and pyruvate [14, 41]. These results indicate that acetogens can perform glycolysis and ferment pyruvate to produce acetate while releasing reducing equivalents.

Only 14 of the 75 genomes harbored genes encoding formate dehydrogenase (FDH; Fig. 4 and Supplementary File S7). These 14 genomes were mostly assigned to *Lachnospirales*. FDH catalyzes CO_2_ reduction to formate as the first step of the methyl branch of the reductive acetogenesis pathway [11, 63, 64]. The other 61 genomes lacked the genes encoding FDH (Fig. 4 and Supplementary File S7), in line with the studies that FDH-lacking acetogens, such as *Blautia wexlerae* and *Clostridium bovifaecis*, are ubiquitous in the gastrointestinal tract [32, 55]. Given that these acetogens inhabit formate-rich environments, they may import formate produced by other bacterial species and utilize it as a substrate for the WL pathway, in an example of metabolic cross-feeding [33], evidenced by 572 genomes encoding formate–nitrite transporter gene (*fcoA* [65] and *nirC* [66]) in this studied dataset (Supplementary File S7). The other way to obtain formate is from organic substrates fermented via glycolysis to pyruvate, which is then degraded to acetyl-CoA and formate by PFL. This possibility is supported by the presence of genes encoding PFL in 35 of the 75 genomes, predominantly members of *Lachnospirales*. The loss of FDH may be explained by the Black Queen Hypothesis (BQH), which proposes that microorganisms may benefit from reductive evolution via adaptive gene loss [67].

As reductive acetogenesis is well known for the use of H_2_ as the electron donor, we looked for pathways of H_2_ metabolism in the genomes. Of the 75 genomes, 54 genomes harbored genes encoding electron-bifurcating (39), fermentative (37), and sensory (13) [FeFe]-hydrogenases (Fig. 4 and Supplementary File S7), suggesting they can use H_2_ as an energy source and potentially an electron donor. Twenty-five of them were found to possess bifurcating hydrogenases but lacked FDH, indicating their potential to use H_2_ to reduce formate during acetogenesis. The other 21 genomes lacked genes encoding hydrogenases and might derive electrons released through fermentative carbohydrate degradation [68, 69] (Fig. 4 and Supplementary File S7). Indeed, it has been shown that H_2_ oxidation by hydrogenases is not an essential part of the WL pathway and can be replaced by other electron-providing reactions [13]. The Rnf complex was also identified, which couples the WL pathway to the generation of a transmembrane ion gradient that drives ATP synthesis [11, 70]. Altogether, the variation of enzymes involved in carbohydrate degradation, H_2_ metabolism, and energy conservation suggests that acetogens adopt a wide range of metabolic strategies and are metabolically versatile in ruminants. The diverse heterotrophic metabolism could serve as a strategic avenue for acetogens to avoid the competition for H_2_ against hydrogenotrophic methanogenesis.

### Dietary treatments selected acetogenic communities with distinct metabolic features in beef cattle

**Figure 5:**
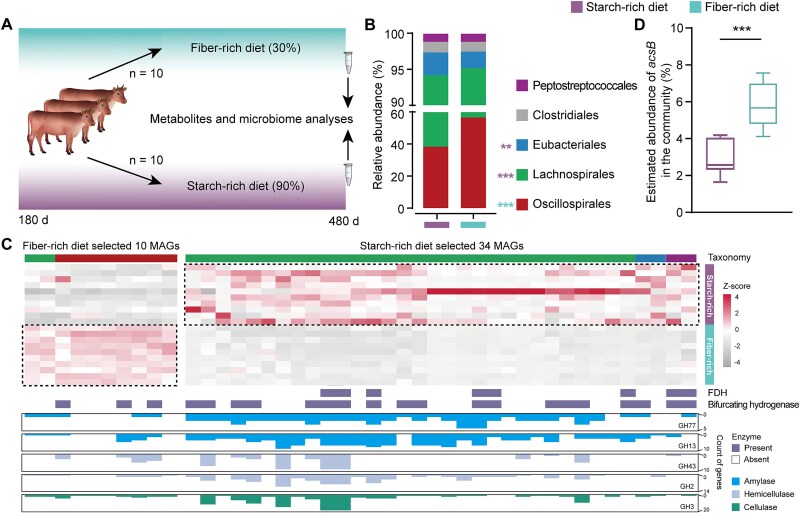
Distinct acetogenic communities with metabolic features selected by contrasting starch-rich and fiber-rich diets. (A) Overview of the animal experiment with concentrate ratio indicated in brackets; (B) order-level acetogenic community composition based on the 75 strain-level genomes; (C) heatmap for differential enrichment of strain-level genomes of putative acetogen based on the Z-score; bar plots below the heatmap represent the gene counts of GH families. Only genomes showing significant differences in relative abundance between the two groups are displayed; (D) the estimated abundance of *acsB* gene in the community. GH, glycoside hydrolase. The Z-score was calculated as: $Z=\frac{X-\mu }{\sigma }$, whereas *X*: Observed value (relative abundance of a genome in an individual sample); *μ*: Population mean relative abundance of that genome across all samples; *σ*: Population standard deviation of the genome’s relative abundance. ^**^P < .01, ^***^P < .001.

Based on the diverse polysaccharide-degradation abilities and energy-conserving strategies of ruminal acetogens, we tested whether a differential acetogenic community in the rumen could be selected by feeding Xiangxi beef cattle with contrasting diets (Fig. 5A, Supplementary File S8, and Table S1). The acetogenic community was altered by the dietary intervention, as indicated by alpha and beta diversity based on the Bray–Curtis dissimilarity matrix (Supplementary Fig. S18). The 34 MAGs of putative acetogen enriched by the starch-rich diet mainly belonged to the orders *Eubacteriales* and *Lachnospirales* (RUG14186 and *Blautia_A*; *P* < .01) and harbored genes encoding amylase (GH77 and GH13; Fig. 5B and C, Supplementary File S9). In contrast, the 10 MAGs enriched by the fiber-rich diet mainly belonged to the orders *Oscillospirales* (RGIG7114; *P* < .01) and encoded hemicellulose- or cellulose-active enzymes (GH43, GH2, and GH3; Fig. 5B and C, Supplementary File S9). Furthermore, the MAGs of putative acetogen selected by the starch-rich diet had a higher copy number of amylase genes than those selected by the fiber-rich diet (6 vs 2 counts/genome; Fig. 5C). These results suggest that the two dietary treatments selected for acetogenic communities with distinct strategies of carbohydrate degradation.

We investigated the differences in the reductive acetogenesis pathways under the two dietary treatments. The fiber-rich diet resulted in a higher proportion of microorganisms encoding the *acsB* marker gene in rumen samples (Fig. 5D, *P* < .05). The enrichment of acetogens in the rumens of animals fed with the fiber-rich diet might be associated with a lower passage rate, resulting in a longer retention time, which provides an appropriate environment for the proliferation of both acetogens and methanogens [45, 71]. All 10 MAGs of putative acetogens enriched by the fiber-rich diet, which were affiliated with *Oscillospirales* and *Lachnospirales*, lacked FDH; among them, only three MAG sharbored group A3 [FeFe] hydrogenases, which are known to mediate electron bifurcating (Fig. 5C). This indicated that fiber-rich diet favored the growth of acetogens that predominantly use electrons from the fermentation of organic matter, and formate plus CO_2_ via the reductive WL pathway, in comparison to the starch-rich diet. Among the 34 MAGs of putative acetogen enriched by the starch-rich diet, 26 MAGs from *Lachnospirales* and *Eubacteriales* lacked FDH, whereas 19 MAGs, primarily belonging to *Lachnospirales*, harbored electron-bifurcating hydrogenases (Fig. 5C). These results indicated that the starch-rich diet favored the growth of acetogens that use H_2_ (self-generated or provided by other H_2_-producing microbes), in comparison to the fiber-rich diet. This enrichment of hydrogenotrophic acetogenic bacteria was consistent with higher dissolved H_2_ concentrations in the rumen of the starch-rich diet (Supplementary Table S4). It has been demonstrated that ruminal reductive acetogenesis can be stimulated when H_2_ concentration is elevated [72–75]. These results suggest that the two contrasting dietary treatments selected for distinct ruminal acetogenic communities that use different electron sources for the WL pathway involved in reductive acetogenesis.

Although acetogens were enriched in the microbiome of animals fed on the fiber-rich diet, the contribution of reductive acetogenesis to total acetate production was lower in the rumen fed with the fiber-rich diet versus the starch-rich diet. For example, the δ^13^C values of individual VFAs in the rumens fed with the fiber-rich diet were higher compared to those from the starch-rich diet (Supplementary Fig. S19). The enrichment of ^13^C in acetate can be attributed to increased background fermentative acetate formation from increased lignocellulosic plant material ingested when fed with the fiber-rich diet. Fiber fermentative degradation favors acetate production over the production of longer-chained fatty acids by starch fermentative degradation [45, 76]. Feeding the fiber-rich diet resulted in a higher molar proportion of acetate and a higher acetate-to-propionate ratio in rumen samples than feeding the starch-rich diet (Supplementary Fig. S20 and Table S4, *P* < .01). The decreased acetate production during carbohydrate fermentation was consistent with the higher dH_2_ concentration in the rumen fed with the starch-rich diet. Further thermodynamic analysis of acetate production from glucose degradation indicated that increasing H_2_ partial pressure can inhibit acetate production by carbohydrate fermentation (due to less negative Gibbs free energy changes) while having little impact on acetate production by heterotrophic acetogenesis in the rumen (Supplementary Fig. S21). These results confirm that the types of carbohydrates ingested, rather than H_2_ partial pressure alone, may be the major driver influencing the contribution of reductive acetogenesis via the WL pathway to total acetate production in the rumen.

### *In vitro* incubations verify distinct capacities of reductive acetogenesis in microbiomes selected by fiber-rich and starch-rich diets

**Figure 6:**
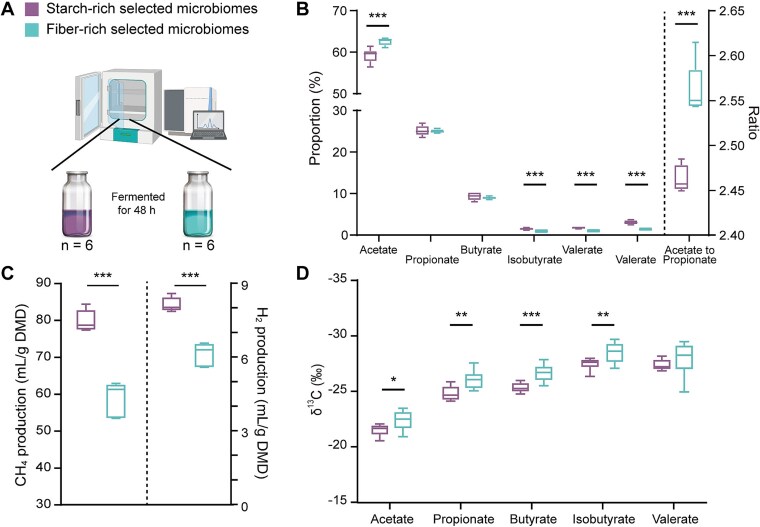
Verification of metabolic activities of microbiomes selected on starch-rich and fiber-rich diets uncovered through 48-hour *in vitro* rumen fermentation. (A) *In vitro* experiment design; (B) molar proportions of individual VFAs and the acetate to propionate ratio; (C) CH_4_ and H_2_ production expressed per gram of dry matter disappearance; (D) stable carbon isotopic fractionation of individual VFAs after 48-hour *in vitro* batch culture. Only the results from fermenting rice straw as the substrate are shown here, and the results from fermenting the starchy substrate or fibrous substrates are shown in Supplementary Table S5. ^*^P < .05, ^**^P < .01, ^***^P < .001, n = 6.

*In vitro* experiment 1 was performed by incubating rumen samples with fibrous, starchy, and rice straw substrates to compare reductive acetogenesis in microbiomes selected by fiber-rich (fiber-selected) versus starch-rich (starch-selected) diets in Xiangxi beef cattle (Fig. 6A). The fiber-selected microbiome produced a greater proportion of acetate and a higher acetate to propionate ratio, with lower CH_4_ and H_2_ production compared to the starch-selected microbiome (Fig. 6B and C and Supplementary Table S5; *P* < .001). Given that the same substrate was incubated during fermentation, the decrease in CH_4_ production in the fiber-selected microbiome can be attributed to an enrichment of acetogens, which would have diverted electrons toward acetate production rather than toward methanogenesis. This aligns with our previous research demonstrating similar metabolic shifts when rice straw was the sole substrate [45]. More detailed investigation, using stable carbon isotope analysis, indicated that the fiber-selected microbiome produced a lower δ^13^C value in each VFA than the starch-selected microbiome (Fig. 6D), which agrees with the enhanced contribution of reductive acetogenesis to acetate production from fiber-selected microbiome versus starch-selected microbiome. During acetate production by classic carbohydrate fermentation, one mole of glucose is oxidized to produce two moles of acetate, two moles of CO_2_, and eight moles of reducing equivalents (C_6_H_12_O_6_ → 2CH_3_COOH + 2CO_2_ + 8[H]) [77]. These reducing equivalents can further be utilized by acetogens via the WL pathway to reduce CO_2_ (or formate plus CO_2_ for acetogens lacking FDH) to produce an additional mol of acetate. This leads to three moles of acetate production by heterotrophic acetogenesis combined with classical fermentation in the same organism (C_6_H_12_O_6_ → 3CH_3_COOH) [11]. Such enhanced heterotrophic acetogenesis in the fiber-rich selected microbiome could be tightly coupled with glycolysis, diverting electrons generated during organic matter oxidation away from H_2_ evolution used for methanogenesis and instead directing them towards VFA synthesis. This would increase acetate production, a product used by ruminants for their nutrition, and decrease CH_4_ production in the rumen. These results confirm the distinct function of acetogenic communities selected by the types of carbohydrates ingested. The enriched acetogenic communities in fiber-rich selected microbiome exhibit an enhanced capacity of reductive acetogenesis for acetate production with reduced rumen methanogenesis.

### DNA stable isotope probing verified the diverse acetogens in the rumen of beef cattle

**Figure 7:**
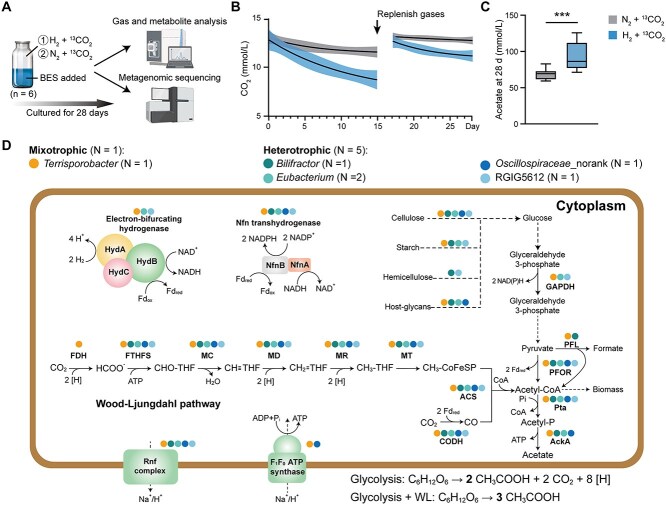
Metabolic pathways of MAGs of putative acetogen enriched through *in vitro* DNA-based stable isotope probing (DNA-SIP). (A) SIP experiment process for enriching ^13^C-labeled acetogenic bacteria through the incubation with H_2_/^13^CO_2_ plus BES, with gas mixture replenished on Day 15. (B) time course of changes in CO_2_ concentration during a 28-day period of incubation; (C) acetate concentrations after a 28-day period of incubation; (D) reconstructed metabolic pathways of six MAGs of putative acetogen obtained from DNA-SIP. The colored circles next to each enzyme or pathway represent the MAGs with the corresponding genus encoding the enzyme. GAPDH, glyceraldehyde-3-phosphate dehydrogenase; PFOR, pyruvate ferredoxin oxidoreductase; Pta, phosphate acetyltransferase; AckA, acetate kinase. [H], reducing equivalent (= 1e^−^ + 1H^+^). ^***^P < .001, n = 6.

*In vitro* experiment 2 was conducted to selectively label and functionally validate acetogens in the rumen microbiota of Xiangxi beef cattle. Rumen inocula were incubated with a methanogen inhibitor while simultaneously tracking metabolic products and microbial DNA incorporation through stable isotope probing (SIP; Fig. 7A). Supplementation with H_2_ gas significantly decreased CO_2_ concentrations and increased acetate production (*P* < .001; 68.5 vs 92.5 mmol/L) compared to the supplementation with N_2_ (Fig. 7B and C), which is consistent with our previous study [38]. These results indicated that the addition of H_2_ as an electron source increased CO_2_ consumption and most probably promoted acetate production via reductive acetogenesis. To illustrate the genomic functions of ^13^CO_2_-consuming microorganisms, the ^13^C-labeled heavy DNA at the end of the incubation was fractionated and sequenced (Supplementary File S10), and six genomes were identified that had the genomic potential to mediate reductive acetogenesis from 480 medium-quality and 110 high-quality bacterial genomes.

One MAG of putative acetogen, SIP_ABG2, belonging to *Terrisporobacter*, harbored all the genes encoding the seven enzymes of the WL pathway, demonstrating the ability to mediate reductive acetogenesis from H_2_ and CO_2_ (Fig. 7D, Supplementary File S11, and Fig. S22). It also possessed genes encoding the group A3 and A4 electron-bifurcating [FeFe]-hydrogenases, ATP synthase, and Rnf complexes, which suggested its capability to convert H_2_ into reductants, to provide electrons for the WL pathway, and to conserve energy in the form of ATP from this reaction. The functions of these hydrogenases have previously been verified in pure cultures of acetogens, including *Acetobacterium woodii*, *Clostridium ljungdahlii*, and *Moorella thermoacetica* [11, 54, 64, 69, 78]. Overall, this SIP experiment further confirmed the existence of hydrogenotrophic acetogens in the rumen of Xiangxi beef cattle, which showed the capacity to reduce CO_2_ to formate using electrons derived from inorganic H_2_.

The other five of the six MAGs of putative acetogen were affiliated with *Eubacterium*, *Bilifractor*, RGIG5612, and *Oscillospiraceae*_norank. They lacked FDH enzymes of the WL pathway, indicating that these acetogens are incapable of autotrophic growth on H₂ plus CO₂ for acetate synthesis (Fig. 7D, Supplementary File S11, and Fig. S22). They instead likely acquired formate through metabolic cross-feeding by other fermentative bacteria. One of them harbored genes encoding PFL, indicating the capacity of producing formate through the pyruvate formate-lyase reaction [33, 79]. Three of the five MAGs were predicted to encode electron-bifurcating hydrogenases (group A3 and A4 [FeFe] hydrogenases), indicating that these formate-dependent putative acetogens may obtain electrons from H_2_ for the reduction of formate in the methyl branch as well as CO_2_ in the carbonyl branch of the WL pathway. These findings confirm the ecological prevalence of the FDH-lacking acetogens in the rumen of Xiangxi beef cattle, where formate serves as a key intermediate and an abundant substance.

All six MAGs of putative acetogens exhibited genetic potential for carbohydrate metabolism, harboring diverse enzymes for carbohydrate degradation and glycolysis (Fig. 7D, Supplementary File S11, and Fig. S22). Specifically, all six MAGs harbored GH3-encoding genes [80] for cellulose degradation, whereas five of the six MAGs (excluding the *Oscillospriaceae*-affiliated SIP_ABG6) harbored GH13 genes [81] associated with starch hydrolysis. Genomic inventories further identified the widespread presence of host-glycan degradation enzymes (GH18, GH25) and key enzymes involved in energy conservation pathways, including glyceraldehyde-3-phosphate dehydrogenase (GAPDH), PFL, and pyruvate ferredoxin oxidoreductase (PFOR). These metabolic features, together with *in vitro* verification experiments, confirm that heterotrophic acetogens prevail in the rumen of Xiangxi beef cattle and could incorporate electrons derived from organic substrates and formate utilization rather than conventional H₂.

## Conclusions

This comprehensive multifaceted study provides definitive evidence that acetogens are widespread and active in ruminants. Based on the analysis of 2102 ruminant metagenomes obtained from 16 countries, phylogenetically diverse and potentially novel acetogens inhabit ruminants, harboring highly conserved genes coding for reductive acetogenesis. These acetogens show the capacity to degrade complex carbohydrates and utilize various electron donors, indicating metabolic versatility and highlighting their flexibility to use electrons from oxidizing both molecular hydrogen and carbohydrates for reductive acetate formation. This heterotrophic metabolic versatility apparently enables acetogens to coexist with methanogens even under thermodynamically disadvantaged conditions and occupy a flexible niche within the complex rumen ecosystem.

Phylogenetic and metabolic diversity of acetogenic communities was distinctly selected by the types of carbohydrates ingested by Xiangxi beef cattle. A starch-rich diet favored the growth of the acetogens equipped with greater starch-degrading capabilities and use electrons obtained from H_2_, whereas a fiber-rich diet favored the growth of heterotrophic acetogens that use electrons obtained from the fermentation of organic matter. These findings were extended by reanalyzing published metagenomic data from the rumen microbiomes of Holstein dairy cows fed high-forage versus high-grain diets, although the abundance of *acsB*-encoding acetogen widely varies between the cattle fed the different diets (supplementary Note 1 and Fig. S23). The putative acetogens enriched in the fiber-rich group may lack strong cellulolytic capabilities (evidenced by no increase in cellulolytic enzyme gene copy numbers), suggesting that other cellulolytic bacteria can provide electrons and/or glucose to these acetogens, and the underlying mechanisms require further investigation. Collectively, this enrichment of heterotrophic acetogens in the microbiome selected by a fiber-rich diet resulted in increased acetate formation and decreased CH_4_ production, apparently through reducing H_2_ supply for methanogenesis.

Redirecting electron flow into metabolic products that benefit the host rather than leading to energy losses in CH_4_ represents an effective strategy for CH_4_ mitigation. It has the promise to simultaneously reduce the environmental impacts of ruminant farming while increasing dietary energy flow to the animal. To realize this potential, it is essential to re-evaluate the overlooked acetogenic community in the rumen, expanding our understanding of the physiology and functionality of acetogens beyond what is currently known from pure culture works. As hydrogenotrophic acetogens cannot compete for H_2_ with methanogens, our findings point to the prioritization of enhancing hitherto unstudied heterotrophic acetogens with the capacity of using electrons derived from fermenting organic substrates, and a possible role of reductive acetogenesis in ruminant nutrition and its potential for mitigating enteric CH_4_ emissions. This potential is further supported by our *in vitro* experiment, in which a doubling in the proportion of *acsB*-encoding acetogens in the fiber-selected microbiome versus the starch-selected microbiome was concurrent with an average 34% reduction in CH_4_ production. Furthermore, enrichment of acetogens can help improve energy utilization efficiency when inhibitors of methanogenesis are supplemented to ruminants, providing an alternative route for electrons towards a product that can improve animal production performance. Future studies with targeted isolation, anaerobic culture, and physiological characterization are warranted to explore the metabolic traits of ruminal acetogens and their synergistic interactions with other polysaccharide-degrading and fermentative bacteria in the rumen. Strategies for enhancing acetogenic activity in rumen of ruminant livestock need further investigation to address global greenhouse gas emissions, supporting efforts to meet climate targets while enhancing the sustainability of ruminant production systems.

## Supplementary Material

Supplementary_information_wraf183

Supplementary_FileS1_wraf183

Supplementary_FileS2_wraf183

Supplementary_FileS3_wraf183

Supplementary_FileS4_wraf183

Supplementary_FileS5_wraf183

Supplementary_FileS6_wraf183

Supplementary_FileS7_wraf183

Supplementary_FileS8_wraf183

Supplementary_FileS9_wraf183

Supplementary_FileS10_wraf183

Supplementary_FileS11_wraf183

## Data Availability

The 69 strain-level MAGs of putative acetogens have been deposited in Figshare (https://doi.org/10.6084/m9.figshare.27283341). Metagenomic sequences of samples from the animal experiments and the SIP experiment are available at the National Center for Biotechnology Information (NCBI, project numbers PRJNA1065619 and PRJNA1085643). All other data supporting the results of this study are available in the article or supplementary information.
