# Supplementary material for: Metabolic versatility enables acetogens to colonize ruminants with diet-driven niche partitioning
Source: ISME J. 2025 Sep 1;19(1):wraf183. doi: 10.1093/ismejo/wraf183 (PMC12449052; doi:10.1093/ismejo/wraf183)
Supplement: Supplementary_information_wraf183 [file supplementary_information_wraf183.docx]

**Supplementary information for**

**Running title: Heterotrophy of Ruminant Acetogens**

**Metabolic versatility enables acetogens to colonize ruminants with diet-driven niche partitioning**

Qiushuang Li^1,2^, Rong Wang^1^, Xiang Zhou^1,2^, Shuya Li^3^, Shizhe Zhang^1,2^, Xiumin Zhang^1^, Wenxing Wang^1,2^, Jinzhen Jiao^1^, Peter H. Janssen^6^, Emilio M. Ungerfeld^7^, Volker Müller^8^, Ralf Conrad^9^, Chris Greening^5^, Zhiliang Tan^1,2*^, Bo Fu^3,4*^, Min Wang^1, 2*^

^1^ *State Key Laboratory of Forage Breeding-by-Design and Utilization, National Engineering Laboratory for Pollution Control and Waste Utilization in Livestock and Poultry Production, and Hunan Provincial Key Laboratory of Animal Nutritional Physiology and Metabolic Process, Institute of Subtropical Agriculture, Chinese Academy of Sciences, Changsha, Hunan* *410125, China*

^2^ *University of Chinese Academy of Sciences, Beijing* *100049, China*

^3^ *School of Environmental Science and Engineering, Wuxi University, Wux**i, Jiangsu 214105, China*

^4^ *School of Environmental and Ecology, Jiangnan University, Wuxi, Jiangsu 214122, China*

^5^ *Biomedicine Discovery Institute, Department of Microbiology, Monash University, Clayton,* *VIC 3800, Australia*

^6^ *AgResearch Limited, Grasslands Research Centre, Palmerston North, Private Bag 11008, New Zealand.*

^7^ *Centro Regional de Investigación Carillanca, Instituto de Investigaciones Agropecuarias (INIA), Temuco, Vilcún 4880000, Chile*

^8^ *Molecular Microbiology & Bioenergetics, Institute of Molecular Biosciences, Goethe-University Frankfurt, Frankfurt, Max-von-Laue Str. 9, Germany*

^9^ *Max Planck Institute for Terrestrial Microbiology, Marburg, 35043, Germany*

^*^Corresponding authors: Zhiliang Tan, State Key Laboratory of Forage Breeding-by-Design and Utilization, Institute of Subtropical Agriculture, Chinese Academy of Sciences, Yuanda road 644, Changsha, Hunan 410125, China. E-mail: zltan@isa.ac.cn; Bo Fu, School of Environmental Science and Engineering, Wuxi University, Xishan Avenue 333, Wuxi, Jiangsu 214105, China, E-mail: fubo@cwxu.edu.cn; and Min Wang, State Key Laboratory of Forage Breeding-by-Design and Utilization, Institute of Subtropical Agriculture, Chinese Academy of Sciences, Yuanda road 644, Changsha, Hunan 410125, China. E-mail: mwang@isa.ac.cn

Competing interests: All the authors declare they have no competing interests.

**This file includes:**

Supplementary Note 1

Supplementary Material and Methods

Supplementary Figure S1 to S23

Supplementary Table S1 to S5

**Supplementary Information Text**

**Supplementary Figures:**

**Figure S1:** The workflow for identifying metagenome-assembled genomes of putative acetogens in the global collection of metagenomes.

**Figure S2.** The genes encoding the key enzymes of the Wood-Ljungdahl pathway.

**Figure S3.** Genome size, GC, completeness, and contamination of 69 selected MAGs of putative acetogens generated from this study.

**Figure S4.** Protein sequence analysis of formate-tetrahydrofolate ligase encoded by *fhs* genes.

**Figure S5.** Protein sequence analysis of CODH/ACS complex subunit encoded by *acsA* genes.

**Figure S6.** Protein sequence analysis of CODH/ACS complex subunit encoded by *acsC* genes.

**Figure S7.** Protein sequence analysis of CODH/ACS complex subunit encoded by *acsD* genes.

**Figure S8.** Protein sequence analysis of formate dehydrogenase subunit encoded by *fdhA* genes.

**Figure S9.** Protein sequence analysis of formate dehydrogenase subunit encoded by *fdhF* genes.

**Figure S10.** Protein sequence analysis of formate dehydrogenase subunit encoded by *hycB* genes.

**Figure S11.** Protein sequence analysis of iron hydrogenase encoded by *hydA2* genes.

**Figure S12.** Protein sequence analysis of methenyltetrahydrofolate cyclohydrolase subunit encoded by *fchA* genes.

**Figure S13.** Protein sequence analysis of methylenetetrahydrofolate reductase subunit encoded by *metF* genes.

**Figure S14.** Protein sequence analysis of methylenetetrahydrofolate reductase subunit encoded by *metV* genes.

**Figure S15.** Protein sequence analysis of methylenetetrahydrofolate reductase subunit encoded by *rnfC2* genes.

**Figure S16.** Protein sequence analysis of methyltransferase subunit encoded by *acsE* genes.

**Figure S17.** Overview of carbohydrate degradation capabilities of 75 genomes of putative acetogens.

**Figure S18.** Comparison of the acetogenic communities in the rumen of beef cattle fed starch-rich and fiber-rich diets.

**Figure S19.** Comparison of stable carbon isotopic fractionation in individual volatile fatty acids in the rumen of beef cattle fed with starch-rich and fiber-rich diets.

**Figure S20.** Molar proportion of individual volatile fatty acids and the ratio of acetate to propionate in the rumen of beef cattle fed starch-rich and fiber-rich diets.

**Figure S21.** Gibbs free energy changes for the fermentation of glucose at different dissolved H_2_ concentrations.

**Figure S22.** Identification of diverse metabolic features in MAGs of putative acetogens enriched through *in vitro* DNA-based stable isotope probing.

**Figure S23.** Distinct acetogenic communities with metabolic features selected by contrasting high-forage and high-grain diets.

**Supplementary Tables:**

**Table S1.** Ingredients and chemical compositions of starch-rich or fiber-rich diets

**Table S2.** Comparative distribution of *acsB*-harboring MAGs among eight ruminant species

**Table S3.** Genomic details of the reference acetogens used in this study

**Table S4.** Fermentation products in the rumen of beef cattle fed with starch-rich or fiber-rich diet.

**Table S5.** Fermentation end products from the fermentation of three substrates in a 48-h *in vitro* batch culture with rumen microbiomes selected by starch-rich or fiber-rich diets.

**Supplementary Files (Excel files)**

**File S1:** Detail of the 20 studies used in this research.

**File S2:** Taxonomic classification and quality assessment of 29,247 integrated MAGs.

**File S3:** Information of the Wood-Ljungdahl genes in 28,738 bacterial MAGs.

**File S4:** Genome and gene information in 69 MAGs of putative acetogens and six single amplified genomes of pure acetogens.

**File S5:** Pairwise local distance distribution tensor (pLDDT) values of each protein sequence in the WL pathway of 75 genomes of putative acetogens.

**File S6:** Root-Mean-Square Deviation (RMSD) values of each protein sequence in the WL pathway of 75 genomes of putative acetogens.

**File S7:** Information of all key metabolic genes in the 75 genomes of putative acetogens and formate–nitrite transporter genes in our genome dataset.

**File S8:** Details of metagenomic samples in the animal experiment.

**File S9:** The relative abundance of 75 genomes of putative acetogens in samples collected in the animal experiment.

**File S10:** Information of metagenomic samples and MAGs in the SIP experiment.

**File S11:** Genome and gene information in the six MAGs of putative acetogens retrieved from the SIP experiment.

**Supplementary Note 1**

We reanalyzed the metagenomic data from the ruminal microbiomes in Holstein cows fed high-forage and high-grain diets to extend the universality of our results obtained by comparing the dietary effect on Xiangxi beef cattle [1]. Consistent with our results, the alpha and beta diversity of the acetogenic community was altered by the distinct diets. Specifically, Shannon index tended to be higher in the high-grain group (*P* = 0.057), and PCoA revealed distinct clustering of microbial communities between the two groups (*P* = 0.001, *R*^2^ = 0.37; Supplementary Figure S19A-C). Moreover, cows fed with the high-forage diet had a higher proportion of microorganisms encoding the *acsB* marker gene (Supplementary Figure S19D). The 29 MAGs of putative acetogens enriched by the high-grain diet mainly belonged to the order *Lachnospirales*, and harbored genes encoding amylase (GH77 and GH13), whereas 20 MAGs of putative acetogens enriched by the high-forage diet mainly belonged to the orders *Oscillospirales* and encoded hemicellulose- or cellulose-active enzymes (GH43, GH2, and GH3). Furthermore, MAGs of putative acetogens selected by the high-grain diet had a higher copy number of amylase genes than those selected by the high-forage diet (7 vs 2 counts/genome; Supplementary Figure S19E). Among the high-forage-enriched MAGs of putative acetogens, only 2 genomes encoded FDH, whereas 9 harbored electron-bifurcating hydrogenases. In contrast, high-grain-enriched MAGs of putative acetogens included 6 FDH-encoding genomes and 15 genomes with electron-bifurcating hydrogenase (Supplementary Figure S19E).

**Supplementary Material and Methods:**

**Functional annotation**

For carbohydrate degradation, genes encoding carbohydrate-active enzymes were annotated using HMMER [2] and DIAMOND [3] to obtain CAZyme annotations through three approaches integrated in dbCAN3 [4]. The specific parameters are as followed: DIAMOND: E-Value < 1e-102, hits per query (-k) = 1; HMMER (dbCAN): E-Value < 1e-15, coverage > 0.35; HMMER (dbCAN-sub): E-Value < 1e-15, coverage > 0.35. A query was acceptable only if all three approaches (DIAMOND, dbCAN_sub, and dbCAN) reported the same match. For hydrogen metabolism, genes encoding hydrogenases (NiFe-, FeFe-, and Fe-hydrogenases) were identified with HydDB [5] by DIAMOND [3] with an e-value threshold of 1e-50, one maximum target sequence per query, and results were then filtered (length of amino acid > 40 residues, sequence identity > 60%).

**Microbial DNA extraction**

Microbial DNA was extracted following the protocol according to a published method based on repeated sand beating plus column methodology [6, 7], and the extracted DNA was subsequently purified using phenol/chloroform/isopentyl alcohol (25:24:1 vol/vol/vol, Solarbio Co., Shanghai, China). The integrity of the extracted DNA was evaluated through electrophoresis on 0.8% agarose gels, and the DNA concentrations and qualities were determined using an ND-2000 spectrophotometer (NanoDrop Technologies, Wilmington, DE). All DNA samples were stored at −80°C until further analyses.

**Genomic context, conserved motifs, and protein structure identification**

The location information of genes involved in the Wood-Ljungahl pathway, including *acsA*, *fdhA*, *fdhF*, *hydA2*, *hycB*, *fhs*, *folD*, *fchA*, *metF*, *metV*, *rnfC2*, *acsE*, *acsD*, *acsC,* and *acsB*, was extracted from the GFF file of each genome. Their relative positions were calculated and then visualized using gggenes (https://wilkox.org/gggenes/). Sequences for each gene were retrieved from acetogens, and conserved motifs were individually identified by Multiple Em for Motif Elicitation (MEME) [8] with default parameters (-protein -oc -mod zoops -nmotifs 8 -minw 6 -maxw 50 -objfun classic -markov_order 0), followed by manual refinement. The sequence’s logo was created using ggseqlogo [9]. Structural modeling of sequences that were annotated with the same function was performed with AlphaFold2 in ColabFold [10]. The predicted local distance difference test value (pLDDT) and predicted template modeling score (pTM) were employed to assess confidence measures. The model with the highest pLDDT and pTM was selected as the representative for each group and visualized in Pymol (https://pymol.org/2/; v2.6.0). The root mean square difference value (RMSD) was calculated for each pair of structures to evaluate the structural similarity of proteins with the same function.

**Metagenome** **sequencing and bioinformatic analysis**

All samples were sequenced on the HiSeq X System (Illumina, San Diego, CA, USA) with pair-end 150 bp (PE150) mode at Shanghai Biozeron Biological Technology Co. Ltd. Low-quality reads, contaminated adaptors, and host reads (Bos_taurus. UMD3.1 [11] and hg19 [12]) were discarded using the Trimmomatic (http://www.usadellab.org/cms/?page=trimmomatic) and BWA packages (v.0.7.12) [13]. High-quality reads in each sample were aligned against 65 acetogens, together with six single amplified acetogens, using BWA-MEM [14] (v.0.7.17). The gene abundance in the community was estimated as “average gene copies per organism” by dividing the abundance of the gene (in reads per kilobase million, RPKM) by the mean abundance of 14 universal single-copy ribosomal marker genes (in RPKM, obtained from the SingleM v0.13.2 package, <https://github.com/wwood/singlem>) as previous described [15]. The relative abundance of acetogens in each sample was calculated and transformed to gene per million (GPM) [16].

Principal coordinate analysis (PCoA) was performed to reveal the differences in the acetogenic community between samples using the *vegan* package [17] in R based on the Bray-Curtis dissimilarity matrix. Analysis of similarities (ADONIS) was performed to indicate group similarity, and the *P* values were determined based on 999 permutations.

**Estimation of Gibbs free energy changes**

Two reaction pathways of glucose fermentation for acetate production under the conditions prevailing in the rumen: reaction 1, C_6_H_12_O_6_ → 2CH_3_COOH + 2CO_2_ + 4H_2_; reaction 2, C_6_H_12_O_6_ → 3CH_3_COOH. Gibbs free energy changes (△G) for these two reactions, allowing fermentation thermodynamics per reaction to be compared, were calculated under rumen conditions: pH of 6.5, dissolved H_2_ of 0.1-600 uM, acetate of 80 mM, a temperature of 39°C, dissolved CO_2_ of 16 mM, and a glucose concentration of 0.1 mM.

**Isotope ratio measurement**

The C isotopic composition of individual VFA was measured using the GC-IsoLink-MS/IRMS system [18]. The GC-IsoLink-MS/IRMS system consisted of a Trace GC Ultra equipped with a TriPlus autosampler, retrofitted to the interface GC IsoLink II for C/H (Thermo Scientific, Bremen, Germany), hyphenated to a Delta V Advantage isotope ratio mass spectrometer (Thermo Scientific, Bremen, Germany), with a single-quadrupole GC-MS (ISQ Thermo Scientific, Milan, Italy). Individual VFA were first separated using a GC-IsoLink-MS module equipped with a DB-FFAP 30 m × 0.25 mm i.d., 0.25 μm film thickness, capillary column (Agilent, Santa Clara, CA, United States), with helium as carrier gas at a flow of 1.2 mL/min. The programmed operating temperature for analysis was as follows: 60 °C held for 2 min and then ramped to 220°C at 20°C/min, and held at 220°C for 0.5 min. The injection was performed at 250°C in splitless mode (2 min) with an injection volume of 1 μL.

The ion source temperature was 230 °C, and the quadruple was held at 150 °C. The mass spectrometer was operated at 70 eV, and the scan range was 50-500 m/z. Individual VFA were identified through their retention times as compared against reference standards and compared with the NIST library (NIST Standard Reference Database1 A NIST/EPA/NIH Mass Spectral Library (NIST 17) and NIST Mass Spectral Search Program (Version 2.2f). Data were collected using the Isodat 3.0 software (Thermo Fisher Scientific).

Before analysis for C isotopic ratio, dry samples of each diet were ground to a fine powder and enclosed in a tin cup, whereas ruminal liquids were packed in 2-ml sample bottles. These separated samples were then transported to the interface GC IsoLink II at the C module and passed through a combustion reactor at 900°C to oxidize the C to CO_2_ gas, and the C isotopic composition of the CO_2_ produced was analyzed by IRMS. The C isotopic composition for all standards and samples was normalized according to Vienna Pee Dee Belemnite (VPDB). Carbon isotopic composition was reported in the delta notation, and calculated as follows:

δ^13^C = 10^3^(R_sample_/R_standard_ -1)

where R= ^13^C/^12^C of the sample and the standard, respectively.

**Supplementary Figures:**


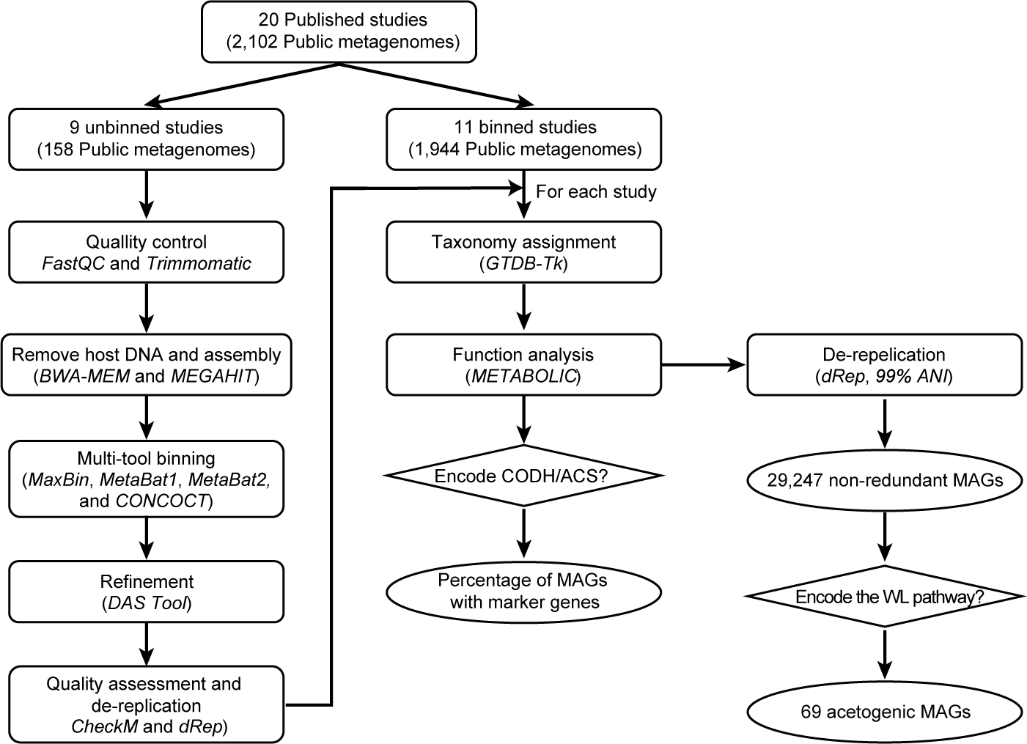


**Supplementary Figure S1.** The workflow for identifying acetogenic metagenome-assembled genomes (MAGs) of putative acetogens in the global collection of metagenomes. The unbinned public metagenomes were *denovo* assembled and binned. All the MAGs from each study were classified and functionally annotated, and the candidate genomes were clustered at 99% average nucleotide identities (ANI). Acetogens were identified by the presence of genes encoding key enzymes of the Wood–Ljungdahl (WL) pathway: formate-tetrahydrofolate ligase, methylenetetrahydrofolate dehydrogenase, methylenetetrahydrofolate cyclohydrolase, methylenetetrahydrofolate reductase, methyltransferase, and CO dehydrogenase/acetyl-CoA synthase.

**
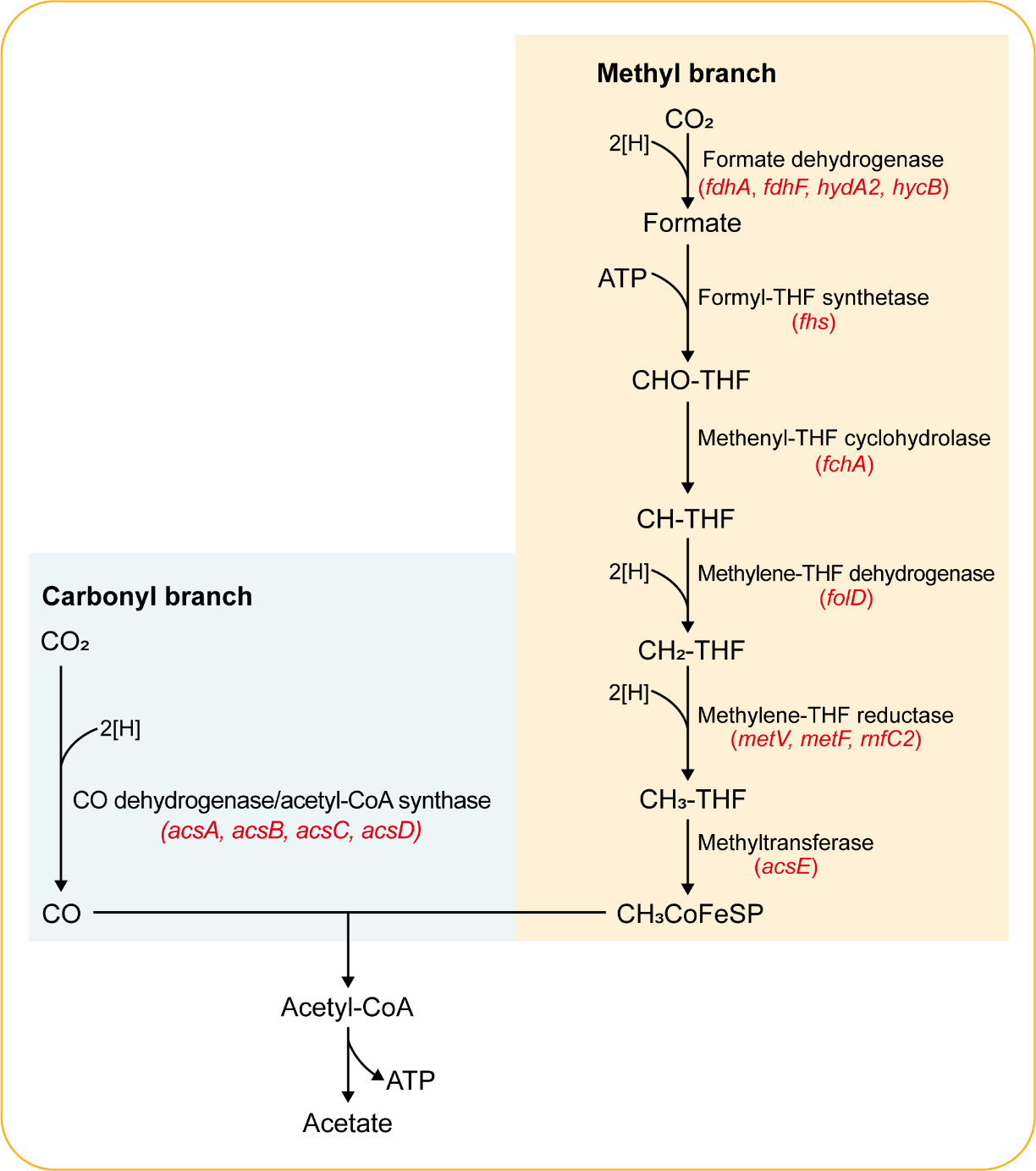
**

**Supplementary Figure S2.** The genes encoding the key enzymes of the Wood-Ljungdahl pathway. *FdhA*, *fdhF*, *hydA2*, *hycB* genes encode formate dehydrogenase; *fhs* genes encode formate-tetrahydrofolate ligase; *fchA* genes encode methenyltetrahydrofolate cyclohydrolase; *folD* genes encode methylenetetrahydrofolate dehydrogenase; *metF*, *metV,* and *rnfC2* genes encode methylenetetrahydrofolate reductase; *acsE* gene encodes methyltransferase; *acsA*, *acsB*, *acsC,* and *acsD* genes encode the carbon monoxide dehydrogenase/acetyl-CoA synthase complex. [H] denotes one reducing equivalent (=1e^−^+1H^+^).


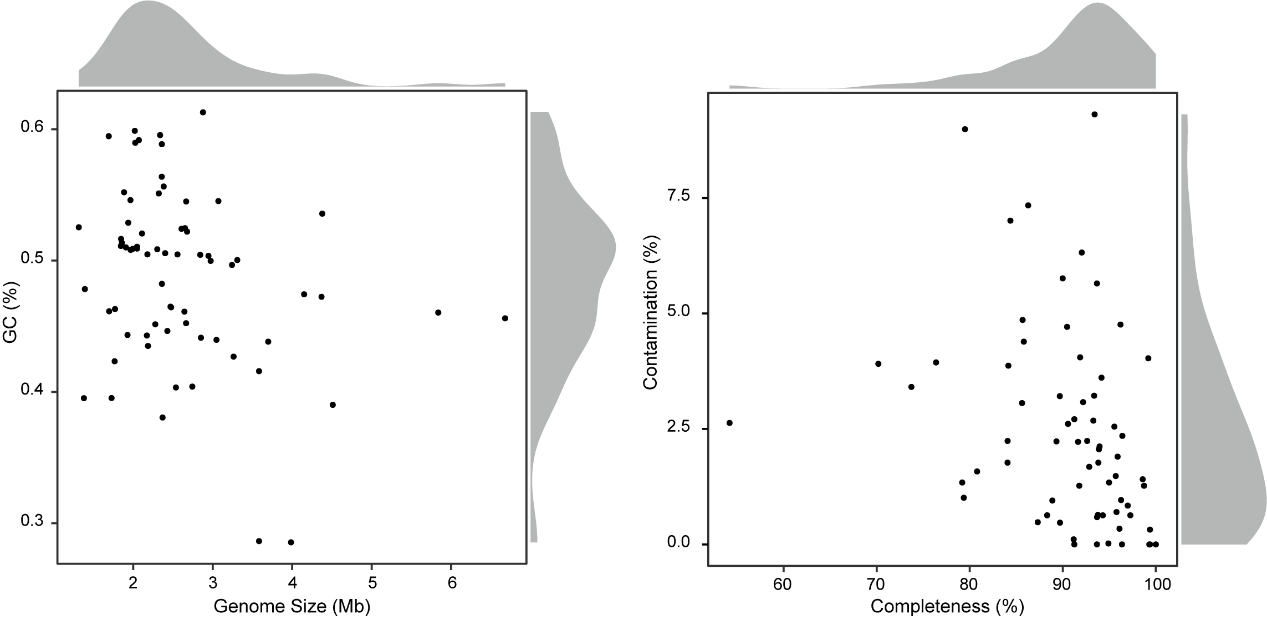


**Supplementary Figure S3.** Genome size, GC, completeness, and contamination of 69 selected MAGs of putative acetogens generated from this study. The grey areas to the top and right of each panel indicate the frequency distributions of points on the opposite axis.

**
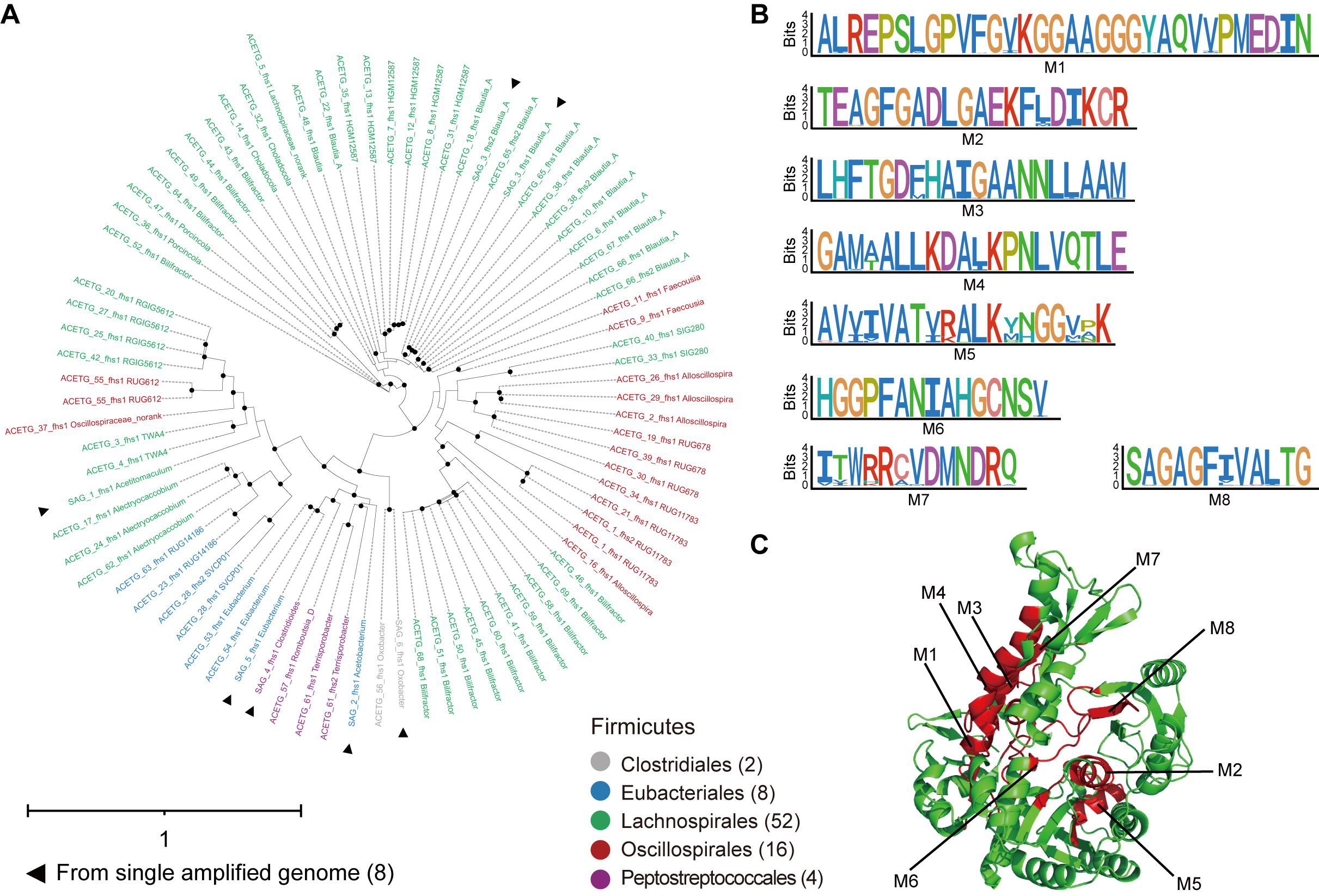
**

**Supplementary Figure S4.** Protein sequence analysis of formate-tetrahydrofolate ligase encoded by *fhs* genes. A. phylogenetic analysis of fhs protein sequences obtained from 75 genomes of putative acetogens. Their phylogenetic affiliations to bacterial orders are indicated by the text color. B, eight amino acid motifs of fhs by Multiple Em for Motif Elicitation (MEME) with default parameters. The size of the graphic character corresponding to each residue is directly proportional to its frequency at that location. C, tertiary structures representing Fhs protein from ACETG_9 MAG (*Faecousia*), which was modeled using AlphaFold2 in ColabFold and subsequently visualized with Pymol. Bootstrap values of >80% are indicated as black circles at the nodes, and the scale bar indicates the average number of substitutions per site. M1 to M8: motif1 to motif8.


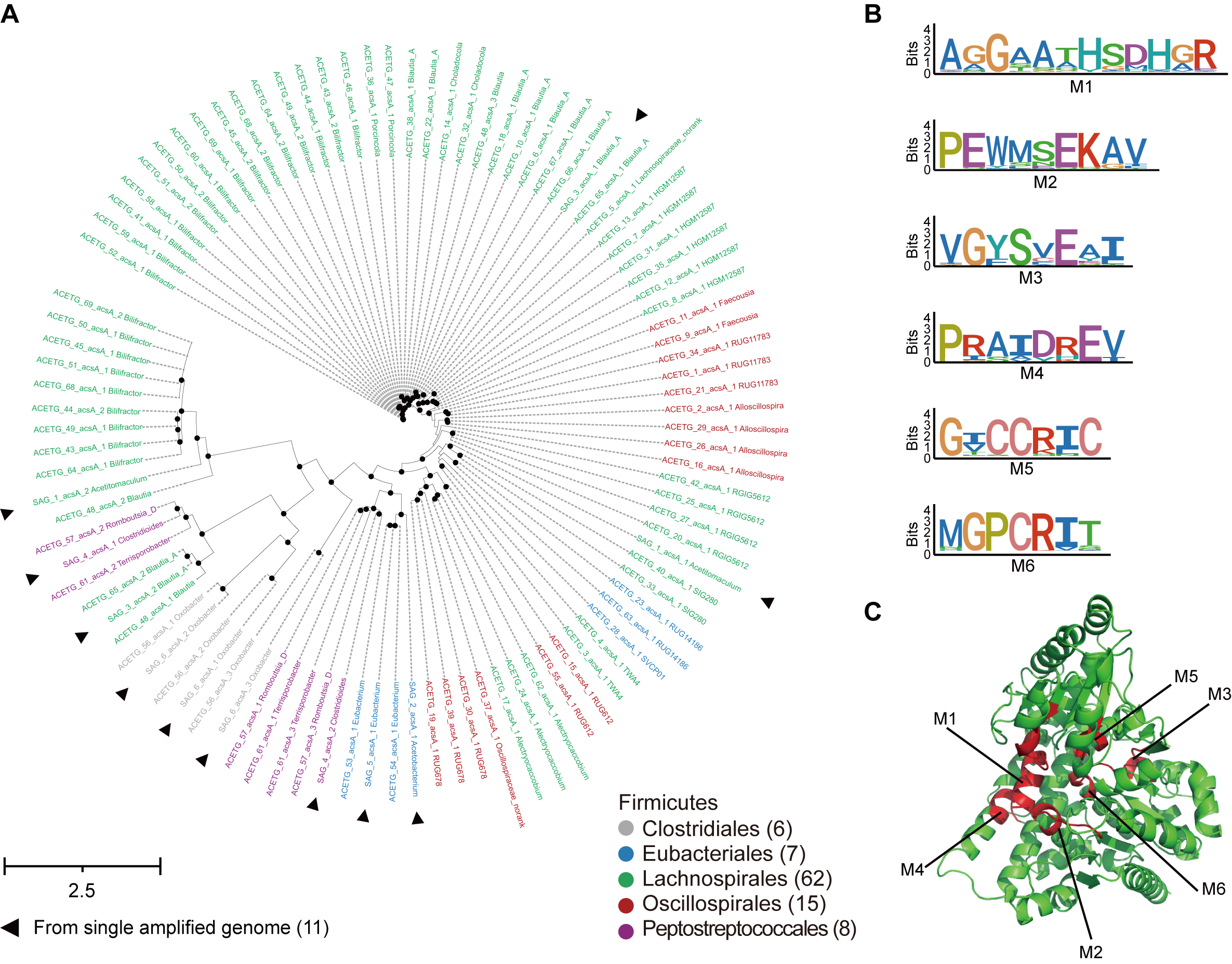


**Supplementary Figure S5.** Protein sequence analysis of CODH/ACS complex subunit encoded by *acsA* genes. A. phylogenetic analysis of acsA protein sequences obtained from 75 genomes of putative acetogens. Their phylogenetic affiliations to bacterial orders are indicated by the text color. B, six amino acid motifs of acsA by Multiple Em for Motif Elicitation (MEME) with default parameters. The size of the graphic character corresponding to each residue is directly proportional to its frequency at that location. C, tertiary structures representing AcsA protein from ACETG_42 MAG (RGIG5612), which was modeled using AlphaFold2 in ColabFold and subsequently visualized with Pymol. Bootstrap values of >80% are indicated as black circles at the nodes, and the scale bar indicates the average number of substitutions per site. M1 to M6: motif1 to motif6.


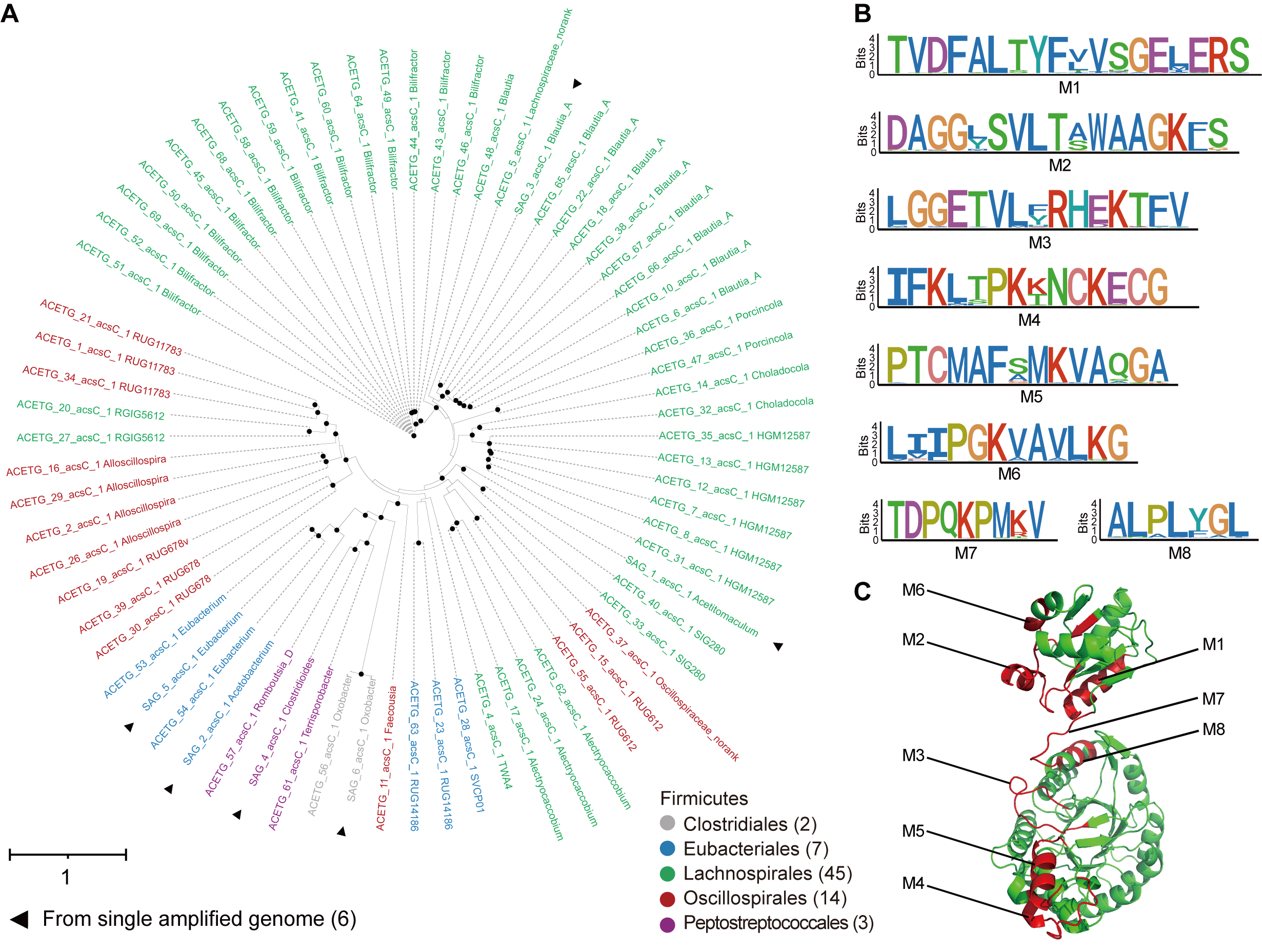


**Supplementary Figure S6.** Protein sequence analysis of CODH/ACS complex subunit encoded by *acsC* genes. A. phylogenetic analysis of acsC protein sequences obtained from 71 genomes of putative acetogens. Their phylogenetic affiliations to bacterial orders are indicated by the text color. B, eight amino acid motifs of acsC by Multiple Em for Motif Elicitation (MEME) with default parameters. The size of the graphic character corresponding to each residue is directly proportional to its frequency at that location. C, tertiary structures representing acsC protein from ACETG_31 MAG (HGM12587), which was modeled using AlphaFold2 in ColabFold and subsequently visualized with Pymol. Bootstrap values of >80% are indicated as black circles at the nodes, and the scale bar indicates the average number of substitutions per site. M1 to M8: motif1 to motif8.


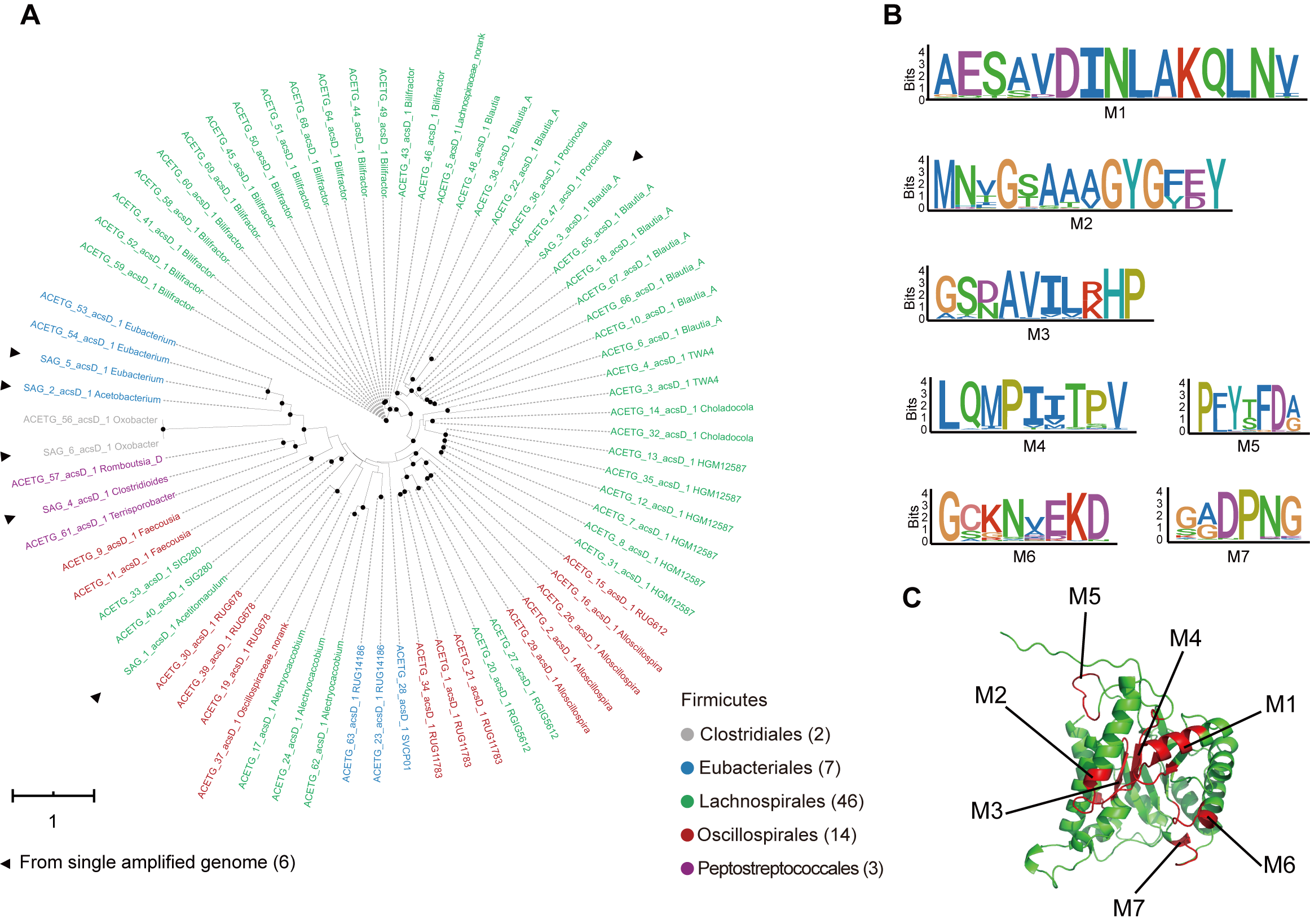


**Supplementary Figure S7.** Protein sequence analysis of CODH/ACS complex subunit encoded by *acsD* genes. A. phylogenetic analysis of acsD protein sequences obtained from 72 genomes of putative acetogens. Their phylogenetic affiliations to bacterial orders are indicated by the text color. B, seven amino acid motifs of acsD by Multiple Em for Motif Elicitation (MEME) with default parameters. The size of the graphic character corresponding to each residue is directly proportional to its frequency at that location. C, tertiary structures representing AcsD protein from ACETG_62 MAG (*Alectryocaccobium*), which was modeled using AlphaFold2 in ColabFold and subsequently visualized with Pymol. Bootstrap values of >80% are indicated as black circles at the nodes, and the scale bar indicates the average number of substitutions per site. M1 to M7: motif1 to motif7.


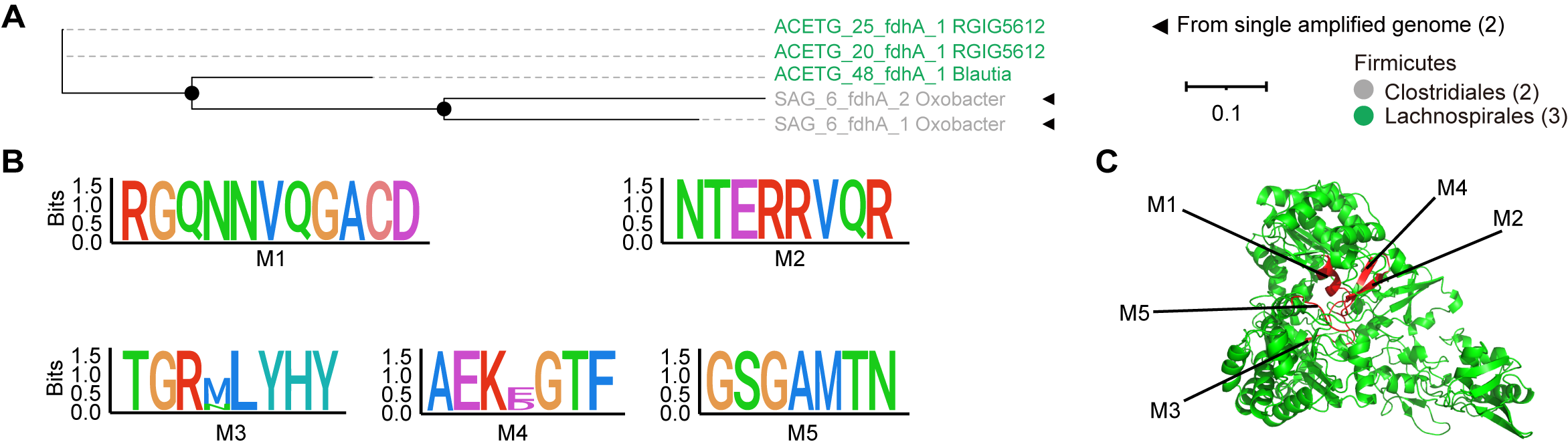


**Supplementary Figure S8.** Protein sequence analysis of formate dehydrogenase subunit encoded by *fdhA* genes. A. phylogenetic analysis of fdhA protein sequences obtained from 4 genomes of putative acetogens. Their phylogenetic affiliations to bacterial orders are indicated by the text color. B, five amino acid motifs of ffdhA by Multiple Em for Motif Elicitation (MEME) with default parameters. The size of the graphic character corresponding to each residue is directly proportional to its frequency at that location. C, tertiary structures representing FdhA protein from ACETG_48 MAG (*Blautia*), which was modeled using AlphaFold2 in ColabFold and subsequently visualized with Pymol. Bootstrap values of >80% are indicated as black circles at the nodes, and the scale bar indicates the average number of substitutions per site. M1 to M5: motif1 to motif5.

**
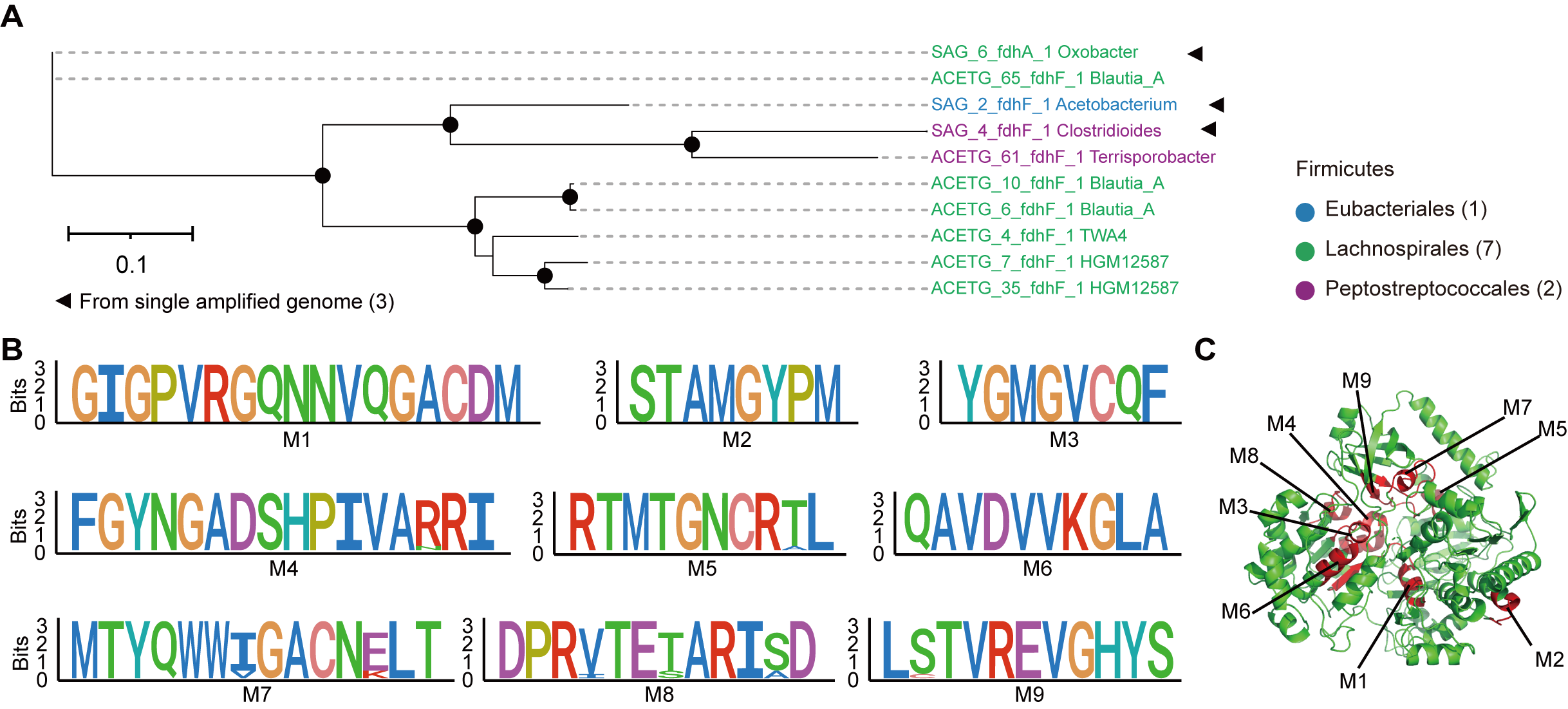
**

**Supplementary Figure S9.** Protein sequence analysis of formate dehydrogenase subunit encoded by *fdhF* genes. A. phylogenetic analysis of fdhF protein sequences obtained from 10 genomes of putative acetogens. Their phylogenetic affiliations to bacterial orders are indicated by the text color. B, nine amino acid motifs of fdhF by Multiple Em for Motif Elicitation (MEME) with default parameters. The size of the graphic character corresponding to each residue is directly proportional to its frequency at that location. C, tertiary structures representing FdhF protein from SAG_2 MAG (*Acetobacterium*), which was modeled using AlphaFold2 in ColabFold and subsequently visualized with Pymol. Bootstrap values of >80% are indicated as black circles at the nodes, and the scale bar indicates the average number of substitutions per site. M1 to M9: motif1 to motif9.


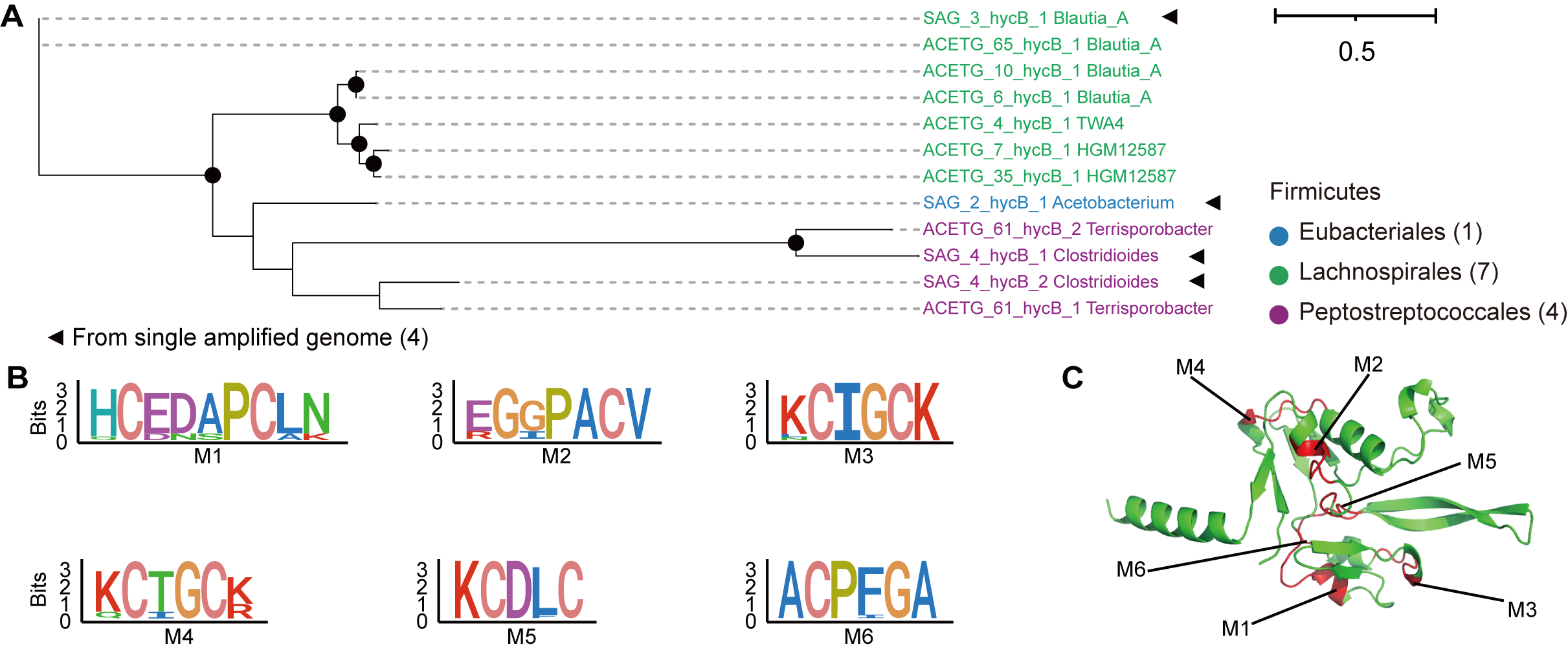


**Supplementary Figure S10.** Protein sequence analysis of formate dehydrogenase subunit encoded by *hycB* genes. A. phylogenetic analysis of hycB protein sequences obtained from 10 genomes of putative acetogens. Their phylogenetic affiliations to bacterial orders are indicated by the text color. B, six amino acid motifs of hycB by Multiple Em for Motif Elicitation (MEME) with default parameters. The size of the graphic character corresponding to each residue is directly proportional to its frequency at that location. C, tertiary structures representing hycB protein from SAG_4 MAG (*Clostridioides*), which was modeled using AlphaFold2 in ColabFold and subsequently visualized with Pymol. Bootstrap values of >80% are indicated as black circles at the nodes, and the scale bar indicates the average number of substitutions per site. M1 to M6: motif1 to motif6.


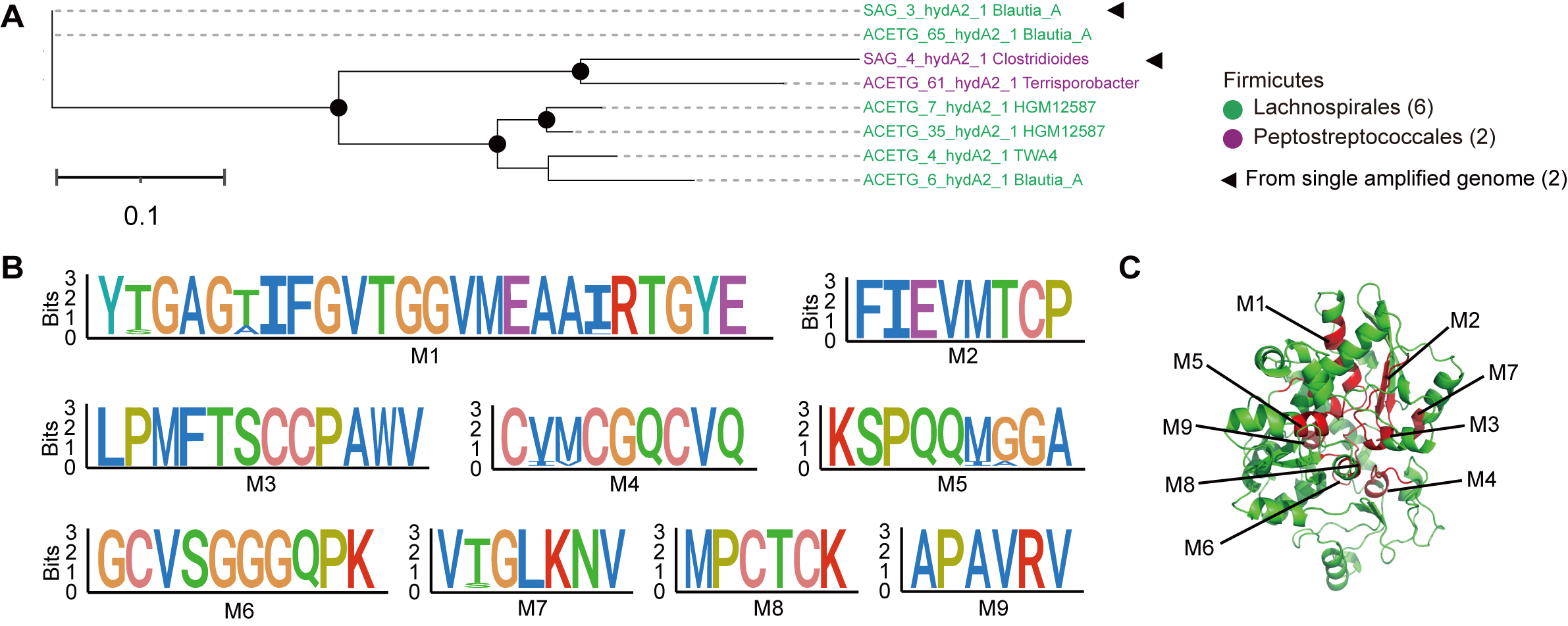


**Supplementary Figure S11.**  Protein sequence analysis of iron hydrogenase encoded by *hydA2* genes. A. phylogenetic analysis of hydA2 protein sequences obtained from 8 genomes of putative acetogens. Their phylogenetic affiliations to bacterial orders are indicated by the text color. B, nine amino acid motifs of hydA2 by Multiple Em for Motif Elicitation (MEME) with default parameters. The size of the graphic character corresponding to each residue is directly proportional to its frequency at that location. C, tertiary structures representing hydA2 protein from ACETG_4 MAG (TWA4), which was modeled using AlphaFold2 in ColabFold and subsequently visualized with Pymol. Bootstrap values of >80% are indicated as black circles at the nodes, and the scale bar indicates the average number of substitutions per site. M1 to M9: motif1 to motif9.

**
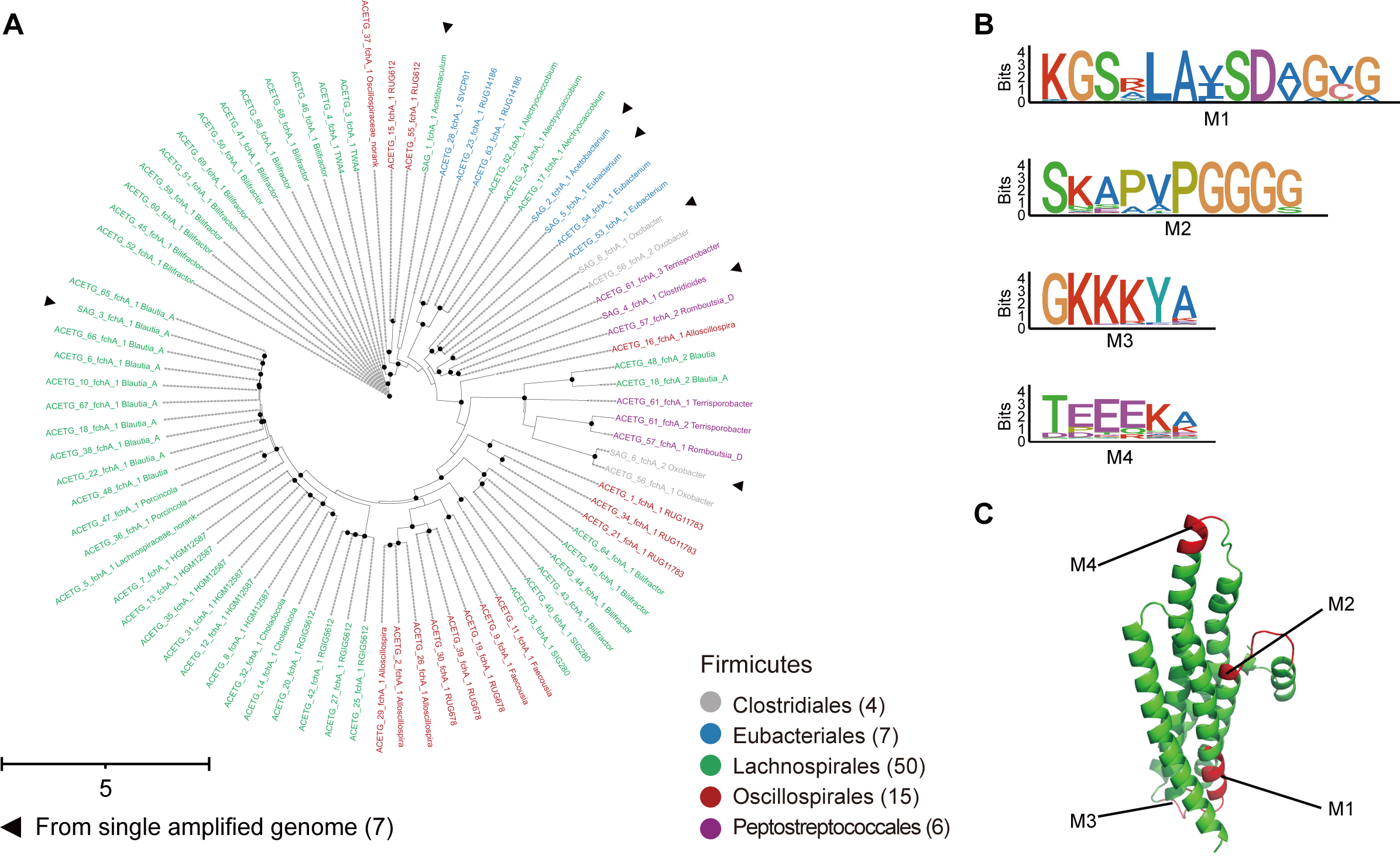
**

**Supplementary Figure S12.** Protein sequence analysis of methenyltetrahydrofolate cyclohydrolase subunit encoded by *fchA* genes. A. phylogenetic analysis of fchA protein sequences obtained from 75 genomes of putative acetogens. Their phylogenetic affiliations to bacterial orders are indicated by the text color. B, four amino acid motifs of fchA by Multiple Em for Motif Elicitation (MEME) with default parameters. The size of the graphic character corresponding to each residue is directly proportional to its frequency at that location. C, tertiary structures representing fchA protein from ACETG_27 MAG (RGIG5612), which was modeled using AlphaFold2 in ColabFold and subsequently visualized with Pymol. Bootstrap values of >80% are indicated as black circles at the nodes, and the scale bar indicates the average number of substitutions per site. M1 to M4: motif1 to motif4.


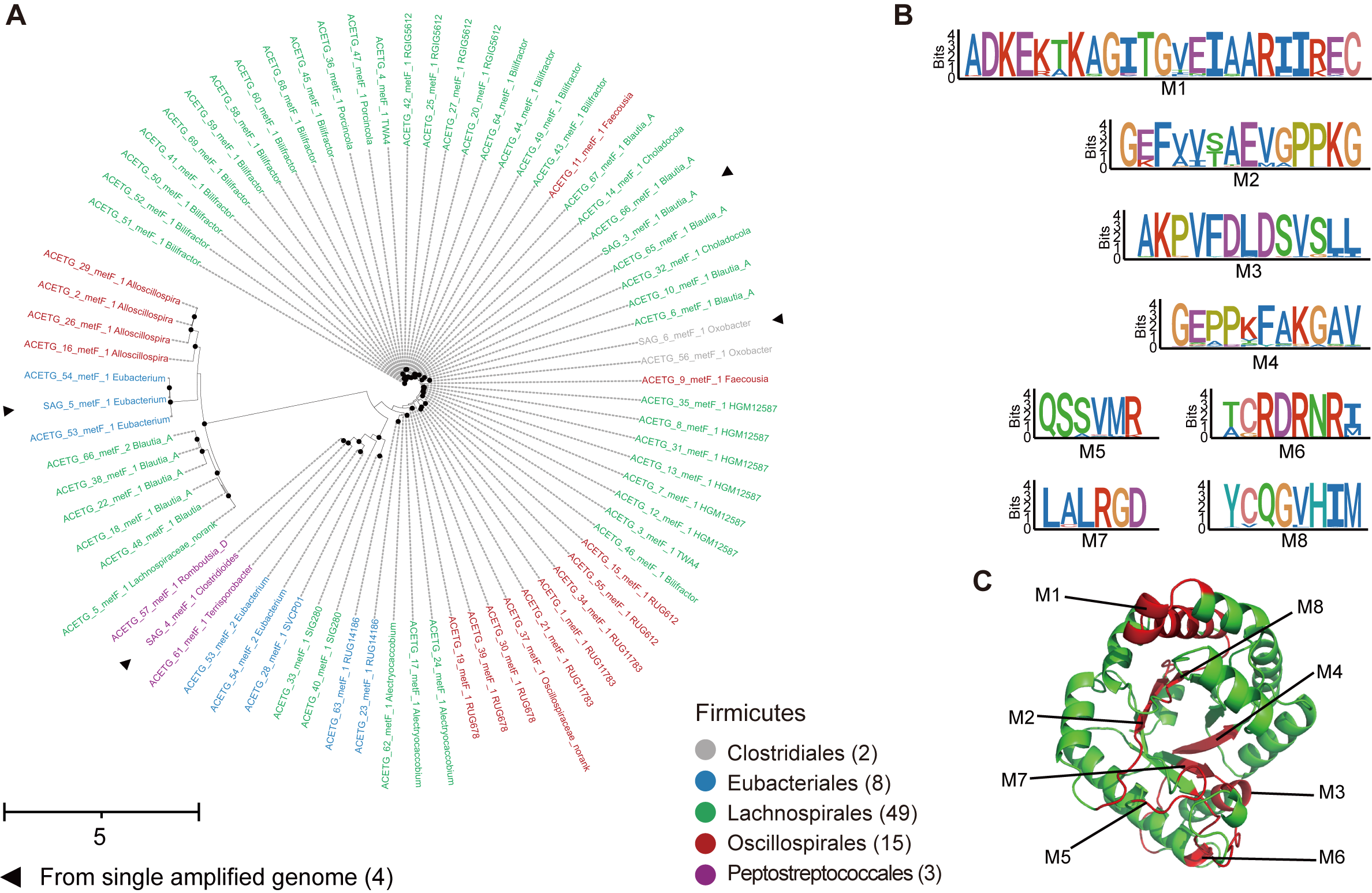


**Supplementary Figure S13.** Protein sequence analysis of methylenetetrahydrofolate reductase subunit encoded by *metF* genes. A. phylogenetic analysis of metF protein sequences obtained from 74 genomes of putative acetogens. Their phylogenetic affiliations to bacterial orders are indicated by the text color. B, eight amino acid motifs of metF by Multiple Em for Motif Elicitation (MEME) with default parameters. The size of the graphic character corresponding to each residue is directly proportional to its frequency at that location. C, tertiary structures representing metF protein from ACETG_42 MAG (RGIG5612), which was modeled using AlphaFold2 in ColabFold and subsequently visualized with Pymol. Bootstrap values of >80% are indicated as black circles at the nodes, and the scale bar indicates the average number of substitutions per site. M1 to M8: motif1 to motif8.


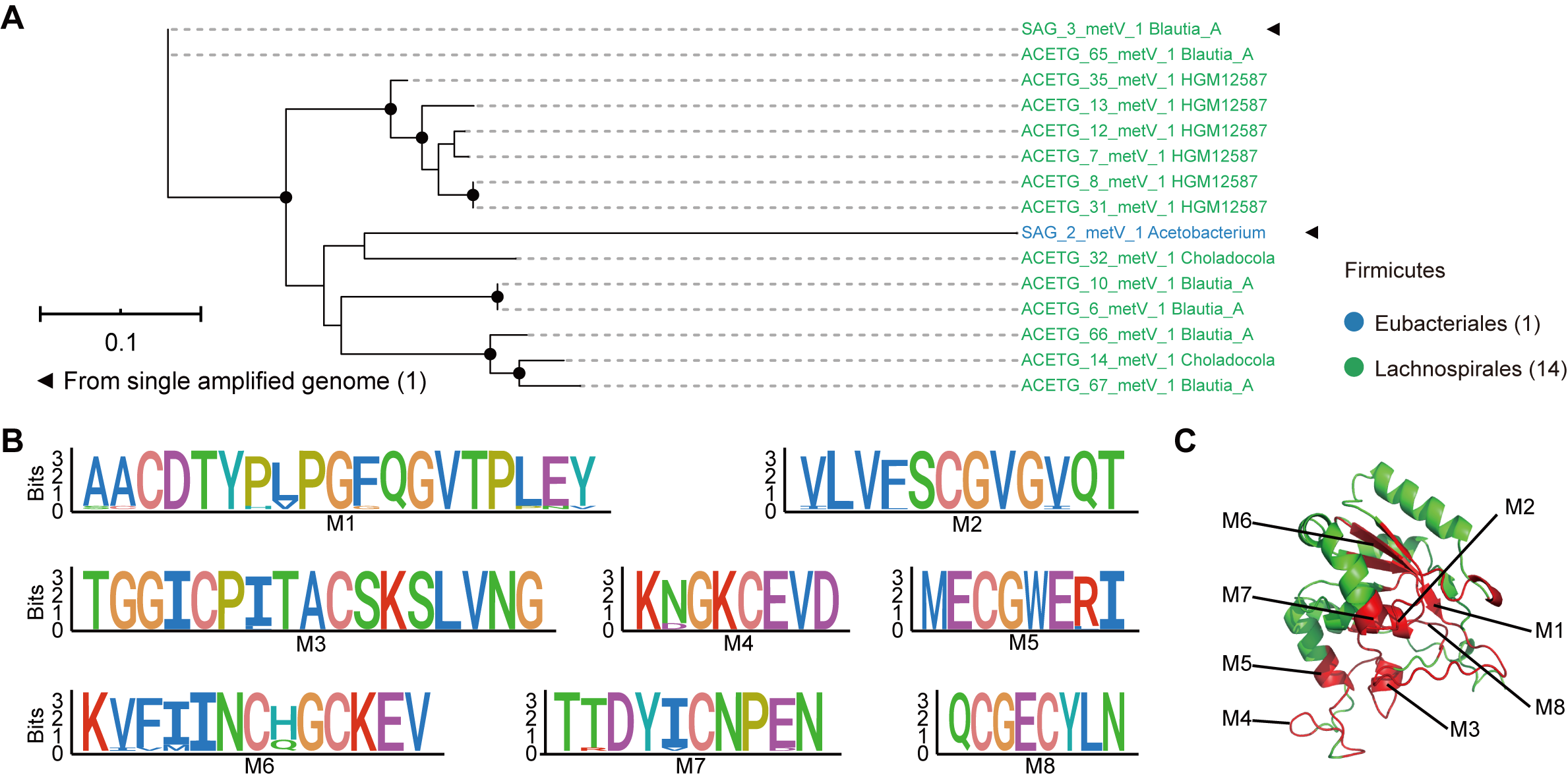


**Supplementary Figure S14.** Protein sequence analysis of methylenetetrahydrofolate reductase subunit encoded by *metV* genes. A. phylogenetic analysis of metV protein sequences obtained from 15 genomes of putative acetogens. Their phylogenetic affiliations to bacterial orders are indicated by the text color. B, eight amino acid motifs of metV by Multiple Em for Motif Elicitation (MEME) with default parameters. The size of the graphic character corresponding to each residue is directly proportional to its frequency at that location. C, tertiary structures representing metV protein from ACETG_6 MAG (*Blautia*_A), which was modeled using AlphaFold2 in ColabFold and subsequently visualized with Pymol. Bootstrap values of >80% are indicated as black circles at the nodes, and the scale bar indicates the average number of substitutions per site. M1 to M8: motif1 to motif8.


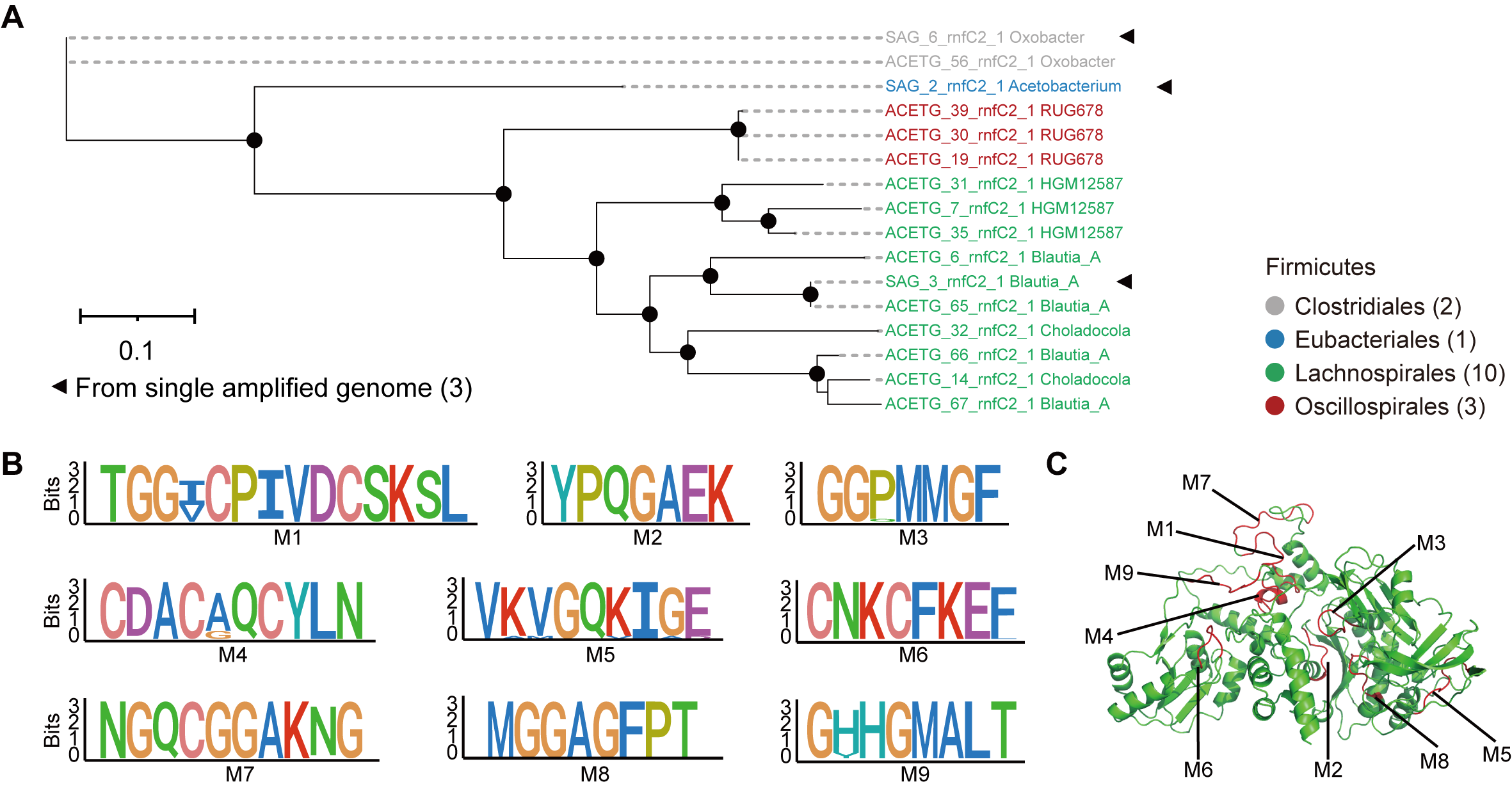


**Supplementary Figure S15.** Protein sequence analysis of methylenetetrahydrofolate reductase subunit encoded by *rnfC2* genes. A. phylogenetic analysis of rnfC2 protein sequences obtained from 16 genomes of putative acetogens. Their phylogenetic affiliations to bacterial orders are indicated by the text color. B, nine amino acid motifs of rnfC2 by Multiple Em for Motif Elicitation (MEME) with default parameters. The size of the graphic character corresponding to each residue is directly proportional to its frequency at that location. C, tertiary structures representing rrnfC2 protein from ACETG_19 MAG (RUG678), which was modeled using AlphaFold2 in ColabFold and subsequently visualized with Pymol. Bootstrap values of >80% are indicated as black circles at the nodes, and the scale bar indicates the average number of substitutions per site. M1 to M9: motif1 to motif9.


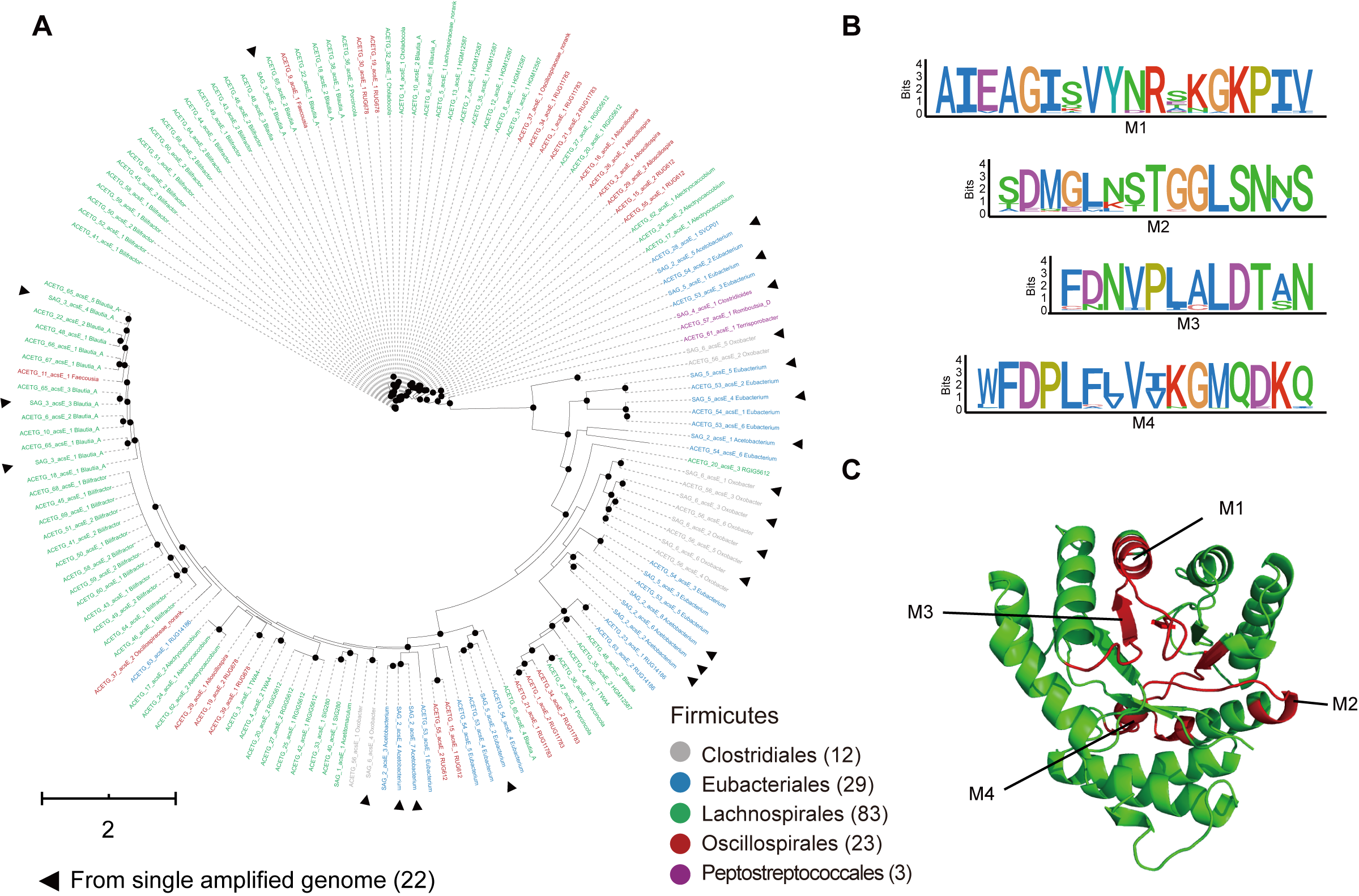


**Supplementary Figure S16.** Protein sequence analysis of methyltransferase subunit encoded by *acsE* genes. A. phylogenetic analysis of acsE protein sequences obtained from 75 genomes of putative acetogens. Their phylogenetic affiliations to bacterial orders are indicated by the text color. B, six amino acid motifs of acsE by Multiple Em for Motif Elicitation (MEME) with default parameters. The size of the graphic character corresponding to each residue is directly proportional to its frequency at that location. C, tertiary structures representing acsE protein from ACETG_35 MAG (RUG11783), which was modeled using AlphaFold2 in ColabFold and subsequently visualized with Pymol. Bootstrap values of >80% are indicated as black circles at the nodes, and the scale bar indicates the average number of substitutions per site. M1 to M4: motif1 to motif4.


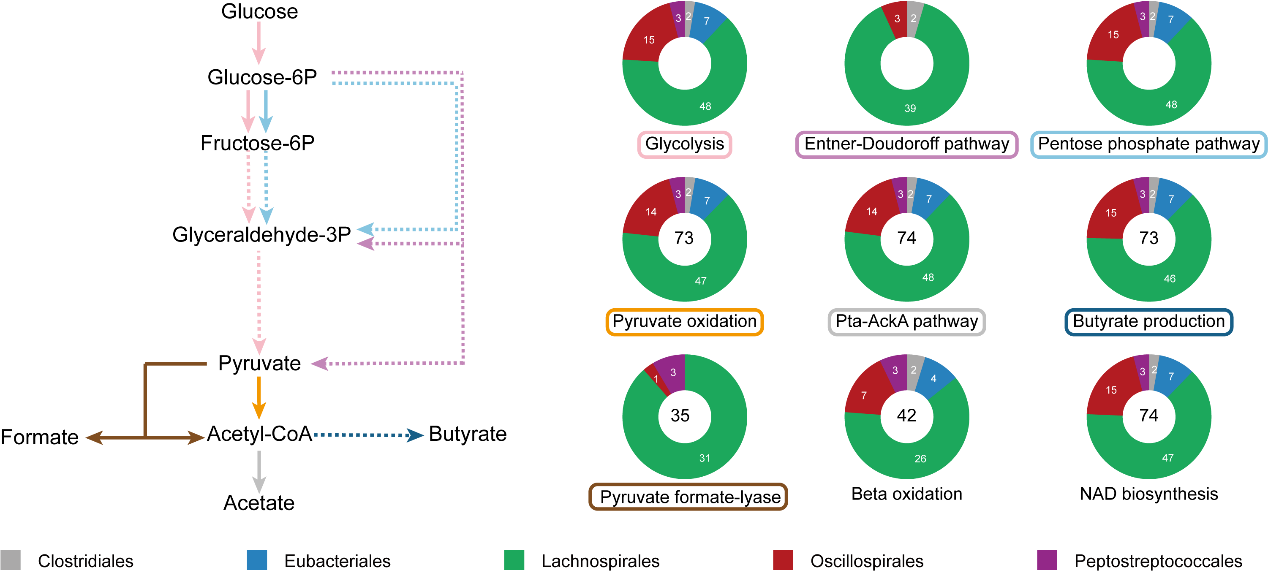


**Supplementary Figure S17.** Overview of carbohydrate degradation capabilities of 75 genomes of putative acetogens. The colors of the lines between the different compounds in the metabolic scheme on the left represent different metabolic pathways. These colors match the borders of the pathway names under the pie charts on the right. The dashed lines denote that the detailed reaction process is omitted. In each pie chart, different colors signify the bacterial order of the MAGs that encode the process, with the number of corresponding MAGs indicated within.

**
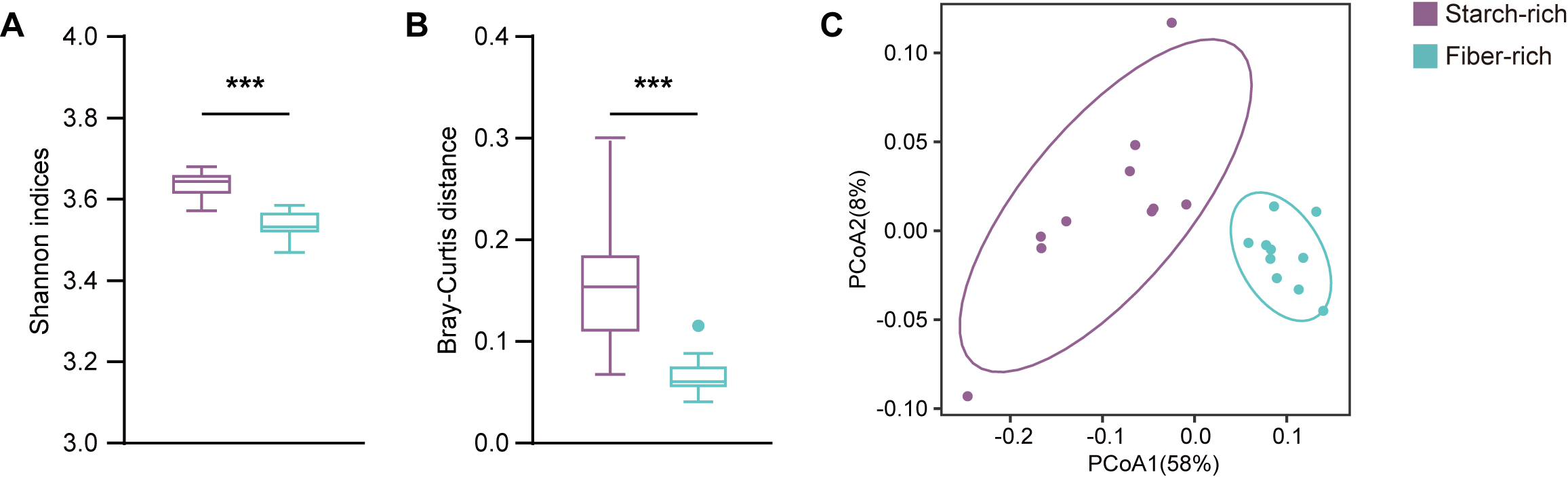
**

**Supplementary Figure S18.** Comparison of the acetogenic communities in the rumen of beef cattle fed starch-rich and fiber-rich diets. A, Shannon indices of acetogenic community; B, Bray-Curtis distances. C, PCoA of the acetogenic community based on Bray-Curtis dissimilarity matrix (PERMANOVA, *P* = 0.001, *R*^2^ = 0.57). ****P* < 0.001, n = 10/group.


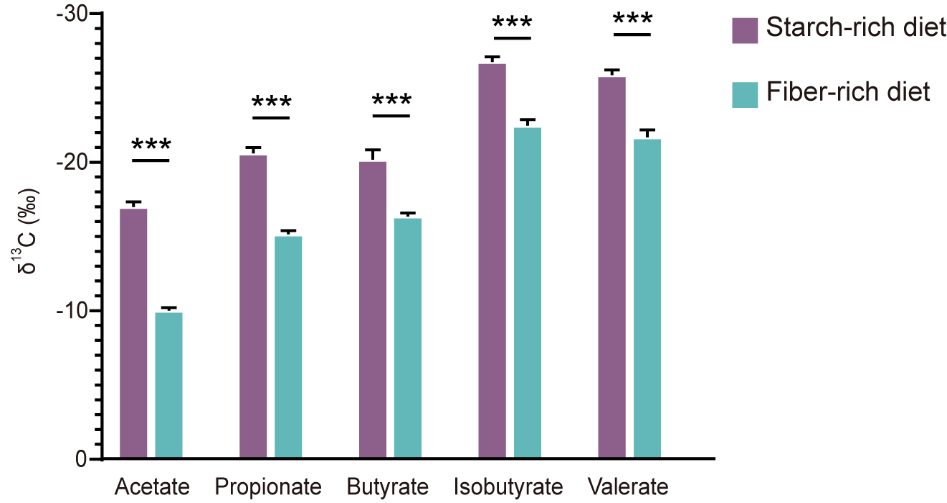


**Supplementary Figure S19.** Comparison of stable carbon isotopic fractionation in individual volatile fatty acids in the rumen of beef cattle fed starch-rich and fiber-rich diets. ****P* < 0.001, n = 10/group.


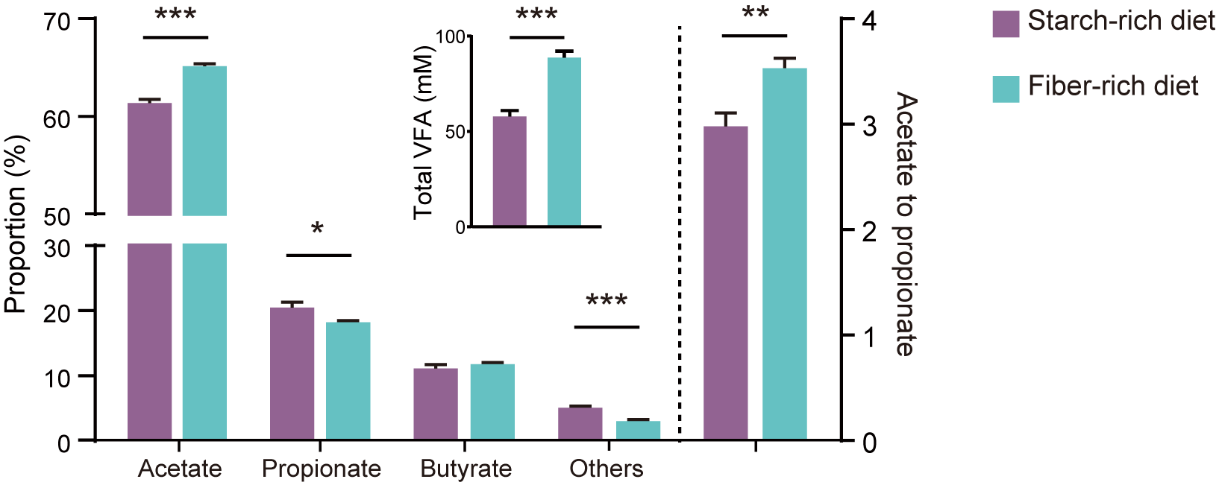


**Supplementary Figure S20.** Molar proportions of individual volatile fatty acids and the ratio of acetate to propionate in the rumen of beef cattle fed starch-rich and fiber-rich diets. **P* < 0.05, ***P* < 0.01, ****P* < 0.001, n = 10/group.


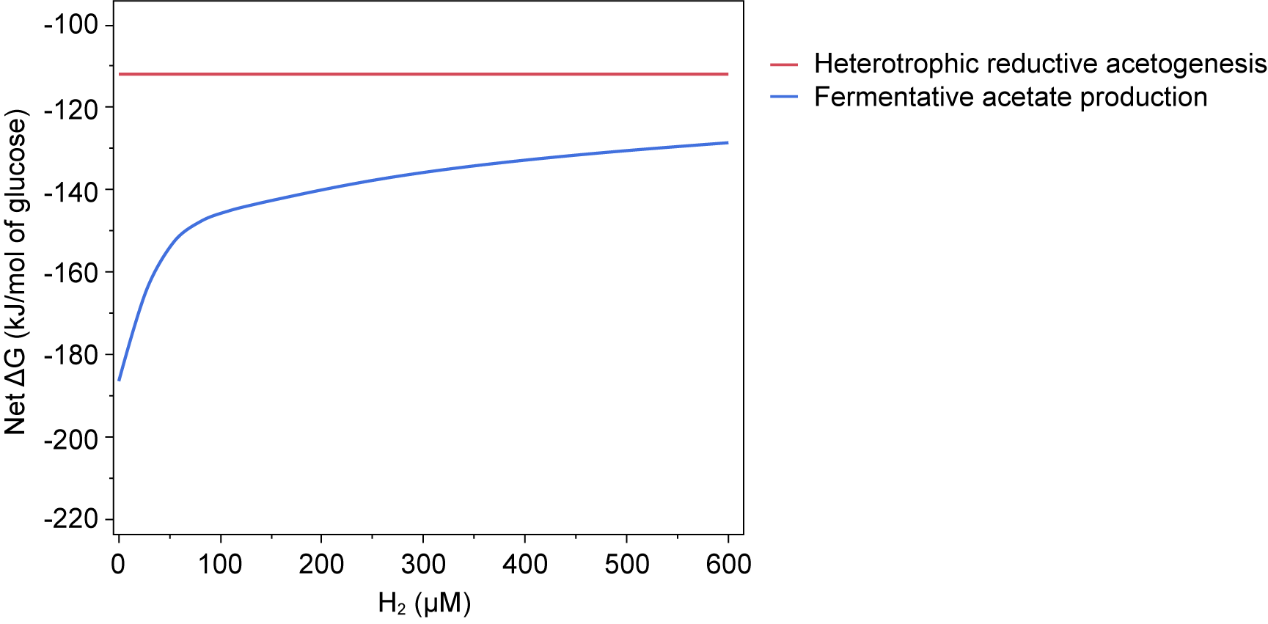


**Supplementary Figure S21.** Gibbs free energy changes (△G) for the fermentation of glucose at different dissolved H_2_ concentrations. The fermentation pathways considered were heterotrophic reductive acetogenesis coupled with the Wood–Ljungdahl (WL) pathway “C_6_H_12_O_6_ → 3CH_3_COOH” (red line), and fermentative acetate production pathway “C_6_H_12_O_6_ → 2CH_3_COOH + 2CO_2_ + 4H_2_” (blue line). The △G values were calculated assuming the following conditions: glucose = 0.1 mM, acetate = 80 mM, CO_2_ = 16 mM, pH = 6.5, *T* = 39^◦^C, H_2_ varied.


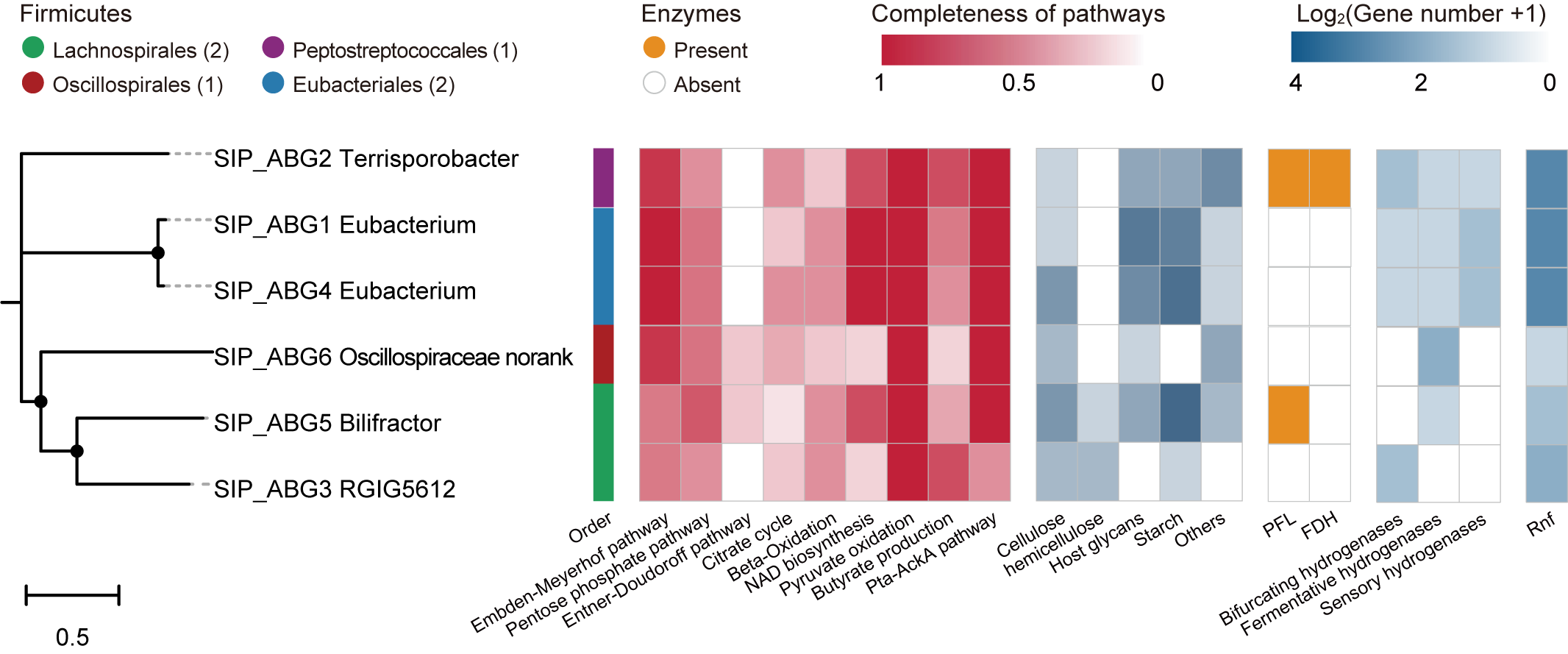


**Supplementary Figure S22.** Identification of diverse metabolic features in MAGs of putative acetogens enriched through *in vitro* DNA-based stable isotope probing (DNA-SIP). These belong to four bacterial orders, indicated by the color key on the top left. The red heatmap indicates the completeness of energy metabolic pathways involved in fermentative glucose degradation; The blue heatmaps indicate the number of genes encoding carbohydrate-degrading enzymes, hydrogenases, and Rnf/Ech complexes; the orange-shaded boxes indicate the presence of genes involved in the Wood–Ljungdahl pathway. Bootstrap values of >70% are indicated as black circles at the nodes, and the scale bar indicates the average number of substitutions per site. Pta-AckA, phosphate acetyltransferase-acetate kinase; PFL, pyruvate formate-lyase; FDH, formate dehydrogenase.

**
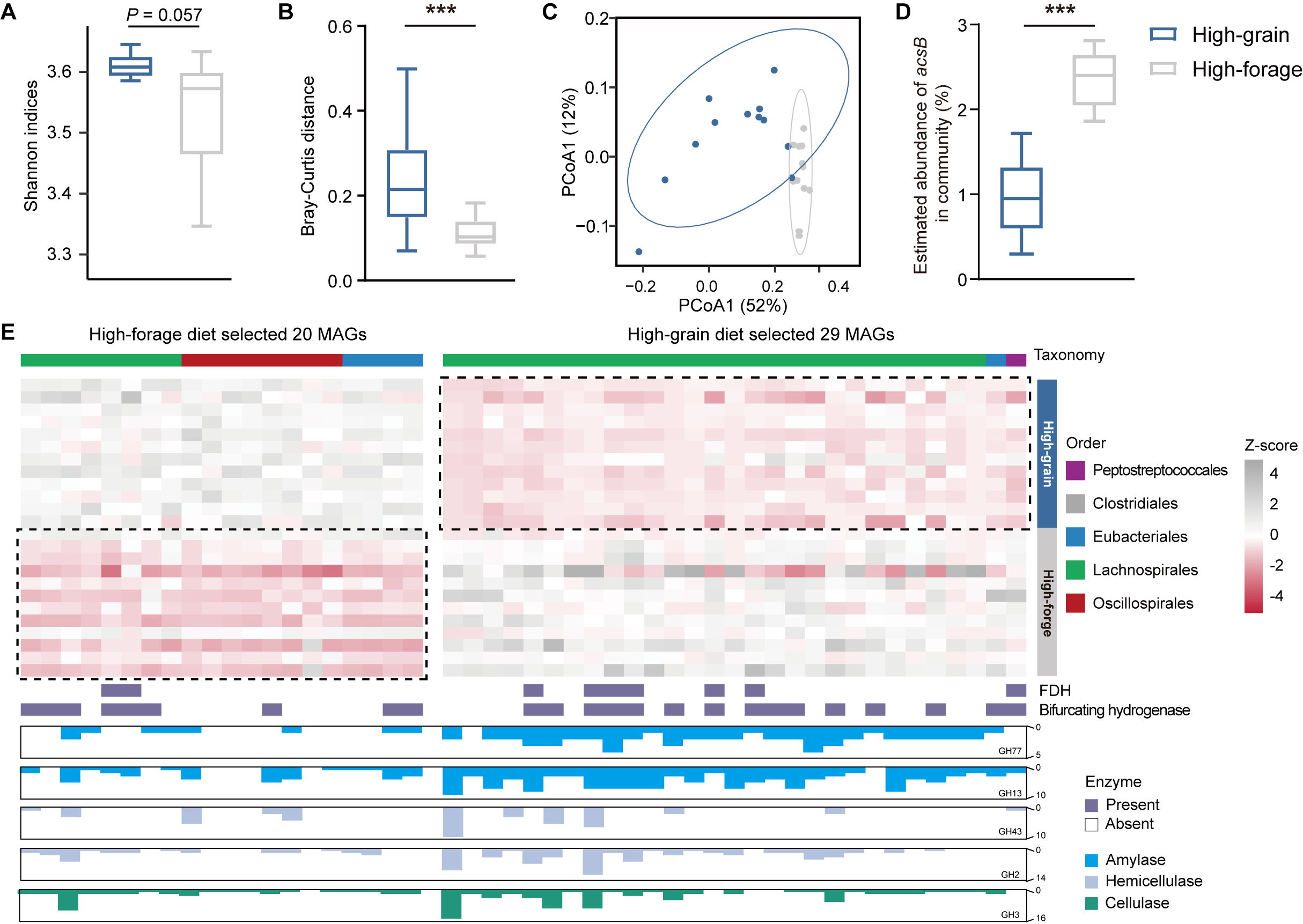
**

**Supplementary Figure S23.** Distinct acetogenic communities with metabolic features selected by contrasting high-forage and high-grain diets. A, Shannon indices of acetogenic community; B, Bray-Curtis distances. C, PCoA of acetogenic community based on Bray-Curtis dissimilarity matrix (PERMANOVA, *P* = 0.001, *R*^2^ = 0.37); D, the established abundance of *acsB* gene in the community; E, heatmap for differential enrichment of strain-level MAGs of putative acetogens based on the Z-score; bar plots below the heatmap represent the gene counts of GH families. Only MAGs showing significant differences in relative abundance between the two groups were displayed. ****P* < 0.001, n = 12/group.

**Supplementary Tables:**

**Table S1.** Ingredients and chemical compositions of starch-rich or fiber-rich diets.

| Item | Starch-rich diet | Fiber-rich diet |
| --- | --- | --- |
|  |  |  |
| Ingredient composition (g/kg DM) | | |
| Forage content | 100 | 700 |
| Corn stover silage ^1^ | 0 | 600 |
| Rice straw ^2^ | 100 | 100 |
| Concentrate | 900 | 300 |
| Soyabean meal | 122 | 140 |
| Barley meal | 665 | 45 |
| Wheat bran | 30 | 30 |
| Puffing urea | 8 | 10 |
| Soybean oil | 20 | 20 |
| Premix ^3^ | 55 | 55 |
| Nutrient composition, g/kg DM | | |
| OM | 903 | 920 |
| Ash | 97 | 80 |
| NDF | 219 | 416 |
| Forage NDF | 661 | 528 |
| ADF | 95 | 215 |
| CP | 150 | 142 |
| Starch^2^ | 364 | 96 |
| Starch/NDF | 1.66 | 0.23 |

ADF, acid detergent fiber; CP, crude protein; DM, dry matter; NDF, neutral detergent fiber; OM, organic matter;

^1^ Corn stover silage contained 438 g/kg of DM, 60 g/kg of CP, 606 g/kg of NDF, 265 g/kg ADF, and 72 g/kg starch on DM basis

^2^ Rice straw contained 967 g/kg of DM, 40 g/kg of CP, 661 g/kg of NDF, 396 g/kg of ADF, and 70 g/kg of starch on DM basis.

^3^ The premix (vitamins and microelements) was formulated to provide (per kg of DM): 5 g of NaCl, 10 g of yeast culture, 1 000 000 IU of vitamin A, 100 000 IU of vitamin D, 682 IU of vitamin E, 4364 mg of Zn, 44 mg of Se, 65 mg of I, 1091 mg of Fe, 22 mg of Co, 1364 mg of Mn, and 1091 mg of Cu.

† Mean values from the analysis of three samples.

**Table S2.** Comparative distribution of *acsB*-harboring MAGs among eight ruminant species

| **Species** | **Number of metagenomes** | **MAGs**  **obtained** | **Number**  **encoding acsB** | **Percent**  **encoding acsB (%)** | **Average**  **(%)** | **References** |
| --- | --- | --- | --- | --- | --- | --- |
| Beef cattle | 24 | 523 | 5 | 0.96 | 1.24 | [19] |
|  | 24 | 1200 | 11 | 0.92 |  | [20] |
|  | 18 | 178 | 5 | 2.81 |  | [21] |
|  | 8 | 317 | 1 | 0.32 |  | [22] |
|  | 48 | 932 | 11 | 1.18 |  | [23] |
| Dairy cow | 24 | 169 | 1 | 0.59 | 1.36 | [24] |
|  | 12 | 176 | 2 | 1.14 |  | [25] |
|  | 72 | 718 | 5 | 0.70 |  | [26] |
|  | 2 | 267 | 8 | 3.00 |  | [27] |
| Buffalo | 695 | 4960 | 142 | 2.86 | 2.86 | [28] |
| Camel | 44 | 591 | 5 | 0.85 | 0.85 | [29] |
| Deer | 50 | 3586 | 43 | 1.20 | 1.20 | [30] |
| Goat | 60 | 2083 | 42 | 2.02 | 2.02 | [30] |
| Sheep | 16 | 2173 | 9 | 0.41 | 0.72 | [31] |
|  | 10 | 194 | 2 | 1.03 |  | [32] |
| Yak | 50 | 1072 | 8 | 0.75 | 0.75 | [30] |

**Table S3.** Genomic details of the reference acetogens used in this study

| **Name** | **Species** | **Isolation Sources** | **Strain** | **GenBank accession number** | **References** |
| --- | --- | --- | --- | --- | --- |
| SAG_1 | *Acetitomaculum ruminis* | Rumen fluid, steer | DSM 5522 | GCF_900112085.1 | [33] |
| SAG_2 | *Acetobacterium fimetarium* | Cattle manure | DER-2019 | GCF_014284475.1 | [34] |
| SAG_3 | *Blautia schinkii* | Rumen of suckling lamb | DSM 10518 | GCF_000702025.1 | [35] |
| SAG_4 | *Clostridioides difficile* | Rumen, newborn lamb | BR81 | GCF_018885085.1 | [36] |
| SAG_5 | *Eubacterium limosum* | Rumen fluid | SA11 | GCF_000807675.2 | [37] |
| SAG_6 | *Oxobacter pfennigii* | Rumen fluid, steer | DSM 3222 | GCF_001317355.1 | [38] |

**Table S4.** Fermentation products in the rumen of beef cattle fed with starch-rich or fiber-rich diets (n = 10/group).

| Item | 0h | | 2.5h | | | 6h | | | | SEM  SEM | | *P* value | | | | | |
| --- | --- | --- | --- | --- | --- | --- | --- | --- | --- | --- | --- | --- | --- | --- | --- | --- | --- |
|  | Starch-rich | Fiber-rich | Starch-rich | Fiber-rich | Starch-rich | | | Fiber-rich |  | | | | Diet | | Time | Diet × Time | |
| pH | 6.9 | 7.1 | 6.8 | 6.9 | 6.8 | | 7.4 | | 0.045 | | 0.002 | | | 0.003 | | | 0.001 |
| VFA concentration, *m*M | 53.0 | 72.7 | 55.2 | 103.7 | 67.7 | | 92.0 | | 2.864 | | <0.001 | | | <0.001 | | | 0.001 |
| Molar percentage of individual VFA, mol/100 mol | | | | | | | | | | | | | | | | | |
| Acetate | 62.3 | 67.9 | 62.4 | 63.0 | 60.5 | | 65.5 | | 0.360 | | <0.001 | | | <0.001 | | | <0.001 |
| Propionate | 19.9 | 16.7 | 21.3 | 20.6 | 22.0 | | 18.8 | | 0.341 | | <0.001 | | | <0.001 | | | 0.085 |
| Butyrate | 11.4 | 11.7 | 11.2 | 12.5 | 12.3 | | 12.5 | | 0.199 | | 0.137 | | | 0.238 | | | 0.447 |
| Others | 6.4 | 3.6 | 5.1 | 3.8 | 5.2 | | 3.2 | | 0.161 | | <0.001 | | | <0.001 | | | 0.002 |
| Acetate to propionate ratio | 3.2 | 4.1 | 3.0 | 3.1 | 2.8 | | 3.5 | | 0.069 | | <0.001 | | | <0.001 | | | 0.001 |
| Dissolved hydrogen, *μ*M | 17.2 | 0.4 | 21.4 | 3.42 | 25.5 | | 2.5 | | 0.437 | | <0.001 | | | 0.05 | | | 0.321 |

VFA, volatile fatty acids

**Table S5.** Fermentation end products from the fermentation of three substrates in a 48-h *in vitro* batch culture with rumen microbiomes selected by starch-rich or fiber-rich diets (n = 6/group).

| Item | Starchy substrate | | Fibrous substrate | | Rice straw | | SEM  SEM | *P* value | | | |  |
| --- | --- | --- | --- | --- | --- | --- | --- | --- | --- | --- | --- | --- |
|  | Starch-rich | Fiber-rich | Starch-rich | Fiber-rich | Starch-rich | Fiber-rich |  | Diet | Substrate | Diet × Substrate | | |
| VFA concentration, mM | 114.1 | 105.0 | 93.1 | 93.5 | 82.3 | 75.8 | 2.674 | 0.138 | <0.001 | 0.498 | | |
| Molar percentage of individual VFA, mol/100 mol | | | | | | | | | | |  |  |
| Acetate | 52.5 | 55.4 | 55.5 | 58.7 | 59.2 | 62.6 | 0.581 | <0.001 | <0.001 | 0.907 | | |
| Propionate | 25.8 | 25.9 | 25.0 | 25.1 | 25.1 | 25.0 | 0.128 | 0.621 | <0.001 | 0.857 | | |
| Butyrate | 12.6 | 12.7 | 10.6 | 10.2 | 9.3 | 9.0 | 0.277 | 0.396 | <0.001 | 0.788 | | |
| Others | 9.1 | 6.1 | 7.78 | 5.1 | 4.0 | 3.4 | 0.333 | <0.001 | <0.001 | 0.845 | | |
| Acetate to propionate ratio | 2.1 | 2.15 | 2.2 | 2.3 | 2.5 | 2.6 | 0.031 | <0.001 | <0.001 | 0.259 | | |
| Methane production (mL/g) | 52.0 | 44.9 | 45.4 | 26.2 | 40.1 | 31.9 | 1.520 | <0.001 | <0.001 | <0.001 | | |
| Hydrogen production (mL/g) | 5.3 | 4.6 | 4.6 | 2.6 | 4.1 | 3.3 | 0.156 | <0.001 | <0.001 | <0.001 | | |
| Dry matter disappearance (%) | 71.7 | 74.9 | 63.5 | 66.2 | 50.3 | 54.3 | 1.551 | <0.001 | <0.001 | 0.837 | | |
| Methane volume (mL/DMD) | 72.7 | 60.2 | 71.5 | 40.0 | 79.8 | 58.8 | 2.343 | <0.001 | <0.001 | <0.001 | | |
| Hydrogen gas volume (mL/DMD) | 7.4 | 6.2 | 7.3 | 4.0 | 8.1 | 6.2 | 0.240 | <0.001 | <0.001 | <0.001 | | |

VFA, volatile fatty acids; DMD, dry matter disappearance.

**References:**

1. Mu YY, Qi WP, Zhang T et al. Gene function adjustment for carbohydrate metabolism and enrichment of rumen microbiota with antibiotic resistance genes during subacute rumen acidosis induced by a high-grain diet in lactating dairy cows. *J Dairy Sci* 2021;**104**:2087-2105. doi: 10.3168/jds.2020-19118

2. Wheeler TJ, Eddy SR. nhmmer: DNA homology search with profile HMMs. *Bioinformatics* 2013;**29**:2487-2489. doi: 10.1093/bioinformatics/btt403

3. Buchfink B, Xie C, Huson DH. Fast and sensitive protein alignment using DIAMOND. *Nat Methods* 2015;**12**:59-60. doi: 10.1038/nmeth.3176

4. Zheng J, Ge Q, Yan Y et al. dbCAN3: automated carbohydrate-active enzyme and substrate annotation. *Nucleic Acids Res* 2023;**51**:W115-W121. doi: 10.1093/nar/gkad328

5. Sondergaard D, Pedersen CN, Greening C. HydDB: a web tool for hydrogenase classification and analysis. *Sci Rep* 2016;**6**:34212. doi: 10.1038/srep34212

6. Ma ZY, Zhang XM, Wang R et al. Effects of chemical and mechanical lysis on microbial dna yield, integrity, and downstream amplicon sequencing of rumen bacteria and protozoa. *Front Microbiol* 2020;**11**:581227. doi: 10.3389/fmicb.2020.581227

7. Yu ZT, Morrison M. Improved extraction of PCR-quality community DNA from digesta and fecal samples. *Biotechniques* 2004;**36**:808-812. doi: 10.2144/04365st04

8. Bailey TL, Johnson J, Grant CE et al. The MEME Suite. *Nucleic Acids Res* 2015;**43**:W39-W49. doi: 10.1093/nar/gkv416

9. Wagih O. ggseqlogo: a versatile R package for drawing sequence logos. *Bioinformatics* 2017;**33**:3645-3647. doi: 10.1093/bioinformatics/btx469

10. Mirdita M, Schutze K, Moriwaki Y et al. ColabFold: making protein folding accessible to all. *Nat Methods* 2022;**19**:679-682. doi: 10.1038/s41592-022-01488-1

11. Shamimuzzaman M, Le Tourneau JJ, Unni DR et al. Bovine genome database: new annotation tools for a new reference genome. *Nucleic Acids Res* 2020;**48**:D676-D681. doi: 10.1093/nar/gkz944

12. International Human Genome Sequencing C. Finishing the euchromatic sequence of the human genome. *Nature* 2004;**431**:931-945. doi: 10.1038/nature03001

13. Li H, Durbin R. Fast and accurate long-read alignment with Burrows-Wheeler transform. *Bioinformatics* 2010;**26**:589-595. doi: 10.1093/bioinformatics/btp698

14. Chaumeil PA, Mussig AJ, Hugenholtz P et al. GTDB-Tk: a toolkit to classify genomes with the Genome Taxonomy Database. *Bioinformatics* 2019;**36**:1925-1927. doi: 10.1093/bioinformatics/btz848

15. Bay SK, Dong XY, Bradley JA et al. Trace gas oxidizers are widespread and active members of soil microbial communities. *Nat Microbiol* 2021;**6**:246-256. doi: 10.1038/s41564-020-00811-w

16. Shaffer M, Borton MA, McGivern BB et al. DRAM for distilling microbial metabolism to automate the curation of microbiome function. *Nucleic Acids Res* 2020;**48**:8883-8900. doi: 10.1093/nar/gkaa621

17. Dixon P. VEGAN, a package of R functions for community ecology. *J Veg Sci* 2003;**14**:927-930. doi: 10.1111/j.1654-1103.2003.tb02228.x

18. Strojnik L, Camin F, Ogrinc N. Compound-specific carbon and hydrogen isotope analysis of volatile organic compounds using headspace solid-phase microextraction. *Talanta* 2020;**219**:121264. doi: 10.1016/j.talanta.2020.121264

19. Gharechahi J, Vahidi MF, Bahram M et al. Metagenomic analysis reveals a dynamic microbiome with diversified adaptive functions to utilize high lignocellulosic forages in the cattle rumen. *ISME J* 2021;**15**:1108-1120. doi: 10.1038/s41396-020-00837-2

20. Wilkinson T, Korir D, Ogugo M et al. 1200 high-quality metagenome-assembled genomes from the rumen of African cattle and their relevance in the context of sub-optimal feeding. *Genome Biol* 2020;**21**:229. doi: 10.1186/s13059-020-02144-7

21. Malmuthuge N, Liang G, Guan LL. Regulation of rumen development in neonatal ruminants through microbial metagenomes and host transcriptomes. *Genome Biol* 2019;**20**:172. doi: 10.1186/s13059-019-1786-0

22. Wallace RJ, Rooke JA, McKain N et al. The rumen microbial metagenome associated with high methane production in cattle. *BMC Genomics* 2015;**16**:839. doi: 10.1186/s12864-015-2032-0

23. Li F, Hitch TCA, Chen Y et al. Comparative metagenomic and metatranscriptomic analyses reveal the breed effect on the rumen microbiome and its associations with feed efficiency in beef cattle. *Microbiome* 2019;**7**:6. doi: 10.1186/s40168-019-0618-5

24. Pitta DW, Indugu N, Melgar A et al. The effect of 3-nitrooxypropanol, a potent methane inhibitor, on ruminal microbial gene expression profiles in dairy cows. *Microbiome* 2022;**10**:146. doi: 10.1186/s40168-022-01341-9

25. Barrett K, Lange L, Borsting CF et al. Changes in the metagenome-encoded cazymes of the rumen microbiome are linked to feed-induced reductions in methane emission from holstein cows. *Front Microbiol* 2022;**13**:855590. doi: 10.3389/fmicb.2022.855590

26. Lin L, Lai Z, Yang H et al. Genome-centric investigation of bile acid metabolizing microbiota of dairy cows and associated diet-induced functional implications. *ISME J* 2023;**17**:172-184. doi: 10.1038/s41396-022-01333-5

27. Hess M, Sczyrba A, Egan R et al. Metagenomic discovery of biomass-degrading genes and genomes from cow rumen. *Science* 2011;**331**:463-467. doi: 10.1126/science.1200387

28. Tong F, Wang T, Gao NL et al. The microbiome of the buffalo digestive tract. *Nat Commun* 2022;**13**:823. doi: 10.1038/s41467-022-28402-9

29. Gharechahi J, Sarikhan S, Han JL et al. Functional and phylogenetic analyses of camel rumen microbiota associated with different lignocellulosic substrates. *NPJ Biofilms Microbiomes* 2022;**8:**1-14. doi: 10.1038/s41522-022-00309-9

30. Xie F, Jin W, Si H et al. An integrated gene catalog and over 10,000 metagenome-assembled genomes from the gastrointestinal microbiome of ruminants. *Microbiome* 2021;**9**:137. doi: 10.1186/s40168-021-01078-x

31. Shi W, Moon CD, Leahy SC et al. Methane yield phenotypes linked to differential gene expression in the sheep rumen microbiome. *Genome Res* 2014;**24**:1517-1525. doi: 10.1101/gr.168245.113

32. Malik PK, Trivedi S, Kolte AP et al. Effect of an anti-methanogenic supplement on enteric methane emission, fermentation, and whole rumen metagenome in sheep. *Front Microbiol* 2022;**13**:1048288. doi: 10.3389/fmicb.2022.1048288

33. Greening RC, Leedle JA. Enrichment and isolation of *Acetitomaculum* *ruminis*, gen. nov., sp. nov.: acetogenic bacteria from the bovine rumen. *Arch Microbiol* 1989;**151**:399-406. doi: 10.1007/BF00416597

34. Kotsyurbenko OR, Simankova MV, Nozhevnikova AN et al. New species of psychrophilic acetogens - *Acetobacterium* *Bakii* sp-nov, a-*Paludosum* sp-nov, a-*Fimetarium* sp-nov. *Arch Microbiol* 1995;**163**:29-34. doi: 10.1007/bf00262200

35. RieuLesme F, Morvan B, Collins MD et al. A new H_2_/CO_2_-using acetogenic bacterium from the rumen: description of *Ruminococcus* *schinkii* sp nov. *Fems Microbiology Letters* 1996;**140**:281-286. doi: 10.1016/0378-1097(96)00195-4

36. Rieu-Lesme F, Dauga C, Fonty G et al. Isolation from the rumen of a new acetogenic bacterium phylogenetically closely related to *Clostridium* *difficile*. *Anaerobe* 1998;**4**:89-94. doi: 10.1006/anae.1998.0153

37. Genthner BR, Davis CL, Bryant MP. Features of rumen and sewage sludge strains of *Eubacterium* *limosum*, a methanol- and H_2_-CO_2_-utilizing species. *Appl Environ Microbiol* 1981;**42**:12-19. doi: 10.1128/aem.42.1.12-19.1981

38. Krumholz Lr, Bryant Mp. *Clostridium* *pfennigii* sp. nov. uses methoxyl groups of monobenzenoids and produces butyrate. *Int J Syst Bacteriol* 1985;**35**:454-456. doi: 10.1099/00207713-35-4-454
